# Supplementary material for: Identification and validation of serum metabolite biomarkers for endometrial cancer diagnosis
Source: EMBO Mol Med. 2024 Feb 14;16(4):988–1003. doi: 10.1038/s44321-024-00033-1 (PMC11018850; doi:10.1038/s44321-024-00033-1)
Supplement: Supplementary file 1 — Appendix [file 44321_2024_33_MOESM1_ESM.pdf]

Appendix for

Identification and validation of serum metabolite biomarkers for endometrial cancer diagnosis

Wanshan Liu†, Jinglan Ma†, Juxiang Zhang, Jing Cao, Xiaoxiao Hu, Yida Huang, Ruimin Wang, Jiao Wu, Wen Di*, Kun Qian*, Xia Yin*

*Correspondence author. Email: diwen@renji.com, k.qian@sjtu.edu.cn, yinxia@renji.com

†These authors contributed equally to this work

Table of contents

Appendix Figures (1-7)	Page 2-8
Appendix Tables (1-7)	Page 9-15

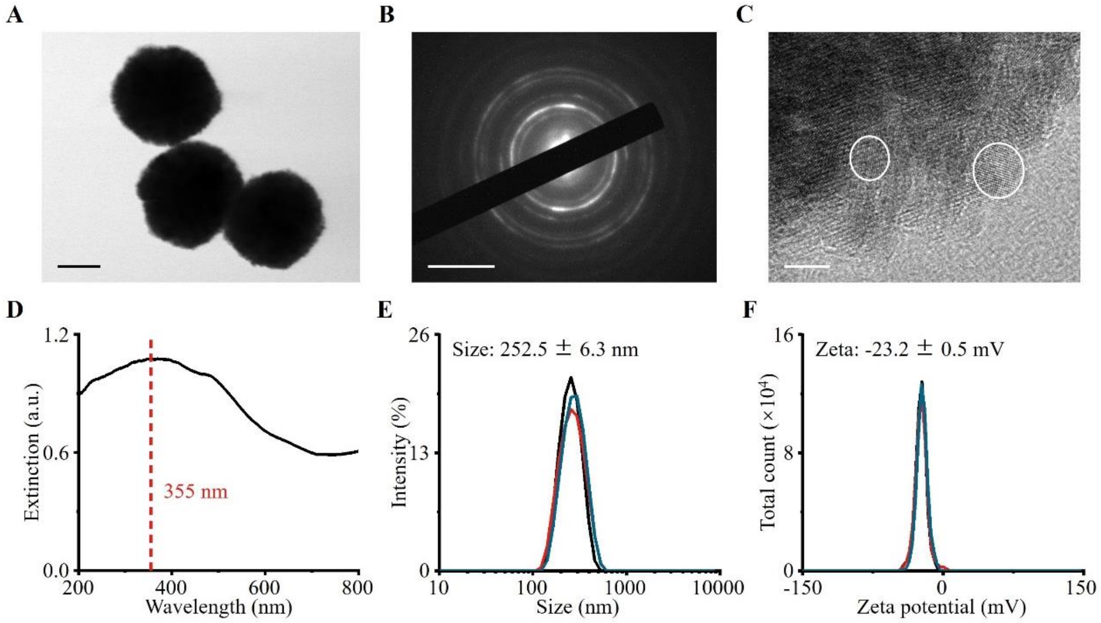

Appendix Figure S1. Characterization of the ferric oxide particles.

A, B. (A) Transmission electron microscopy (TEM) image (scale bar = 100 nm) and **(B)** selected area electron diffraction (SAED) pattern (scale bar = 5 nm^{-1}) of the ferric oxide particles.

C. High-resolution TEM (HRTEM) image displayed the crystal lattice of the ferric oxide particles, marked by white circles. The scale bar was 5 nm.

D. Ultraviolet-visible (UV-Vis) spectrum of the ferric oxide particles showed a strong absorbance at 355 nm.

E, F. (E) Dynamic light scattering (DLS) and **(F)** zeta potential were recorded by 3 independent technical replicates. Data were mean ± SD, $N = 3$ technical replicates.

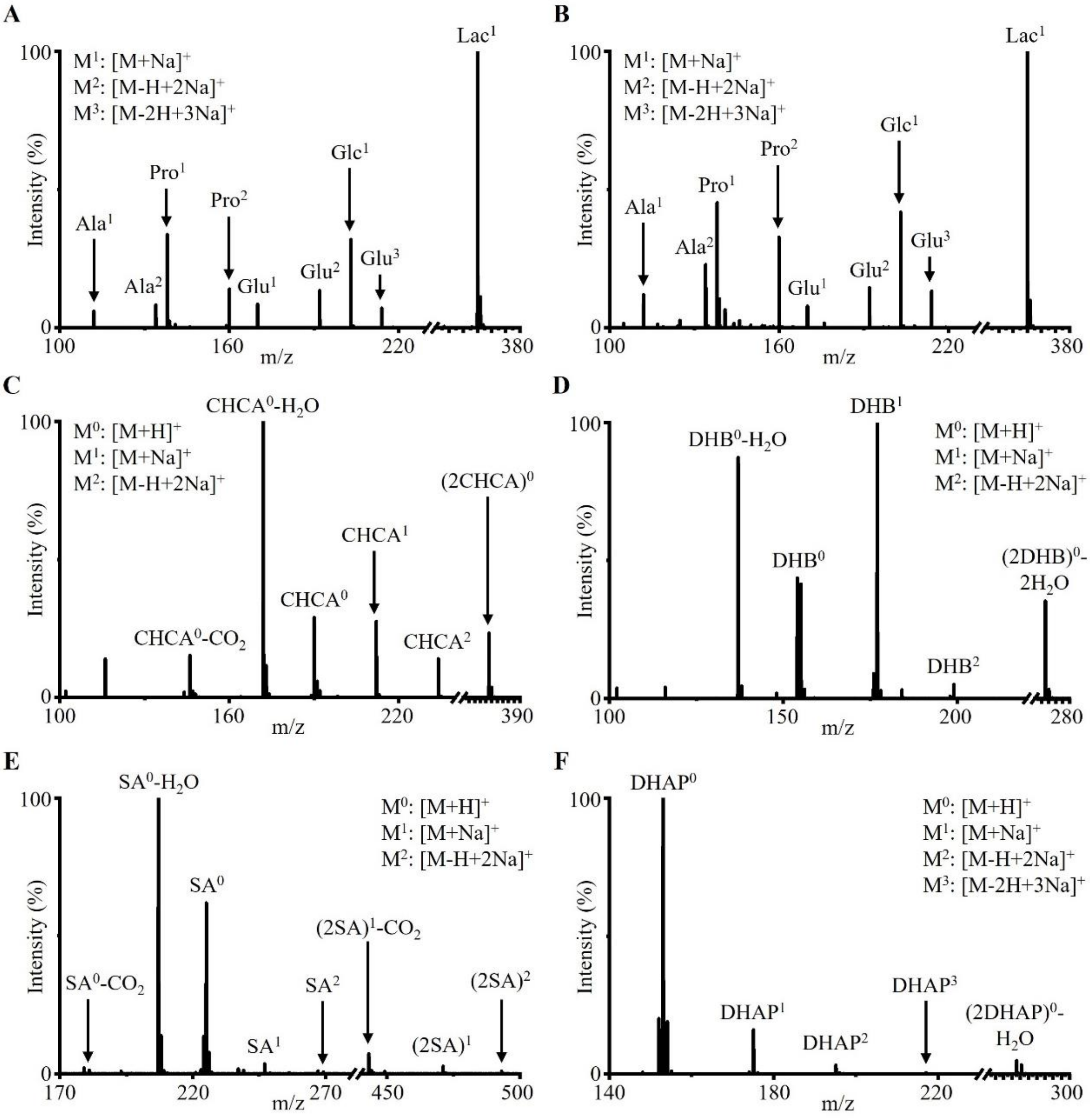

Appendix Figure S2. High salt and protein tolerance of PELDI-MS.

A, B. Typical mass spectrum of the standard sample under **(A)** high salt condition (20 mM Na^+) and **(B)** biofluid-mimic condition (20 mM Na^+ and 10 mg/mL protein) using ferric oxide particles. Alkali metal cation adduction ($[M+Na]^+$, $[M-H+2Na]^+$, and $[M-2H+3Na]^+$) of small metabolites (alaline (Ala), proline (Pro), glutamic acid (Glu), glucose (Glc), and lactose (Lac)) was marked.

C-F. Typical mass spectra of the standard sample under high salt condition (20 mM Na^+) using **(C)** α-cyano-4-hydroxycinnamic acid (CHCA), **(D)** 2,5-dihydroxybenzoic acid (DHB), **(E)** sinapic acid (SA), and **(F)** 2,6-dihydroxyacetophenone (DHAP).

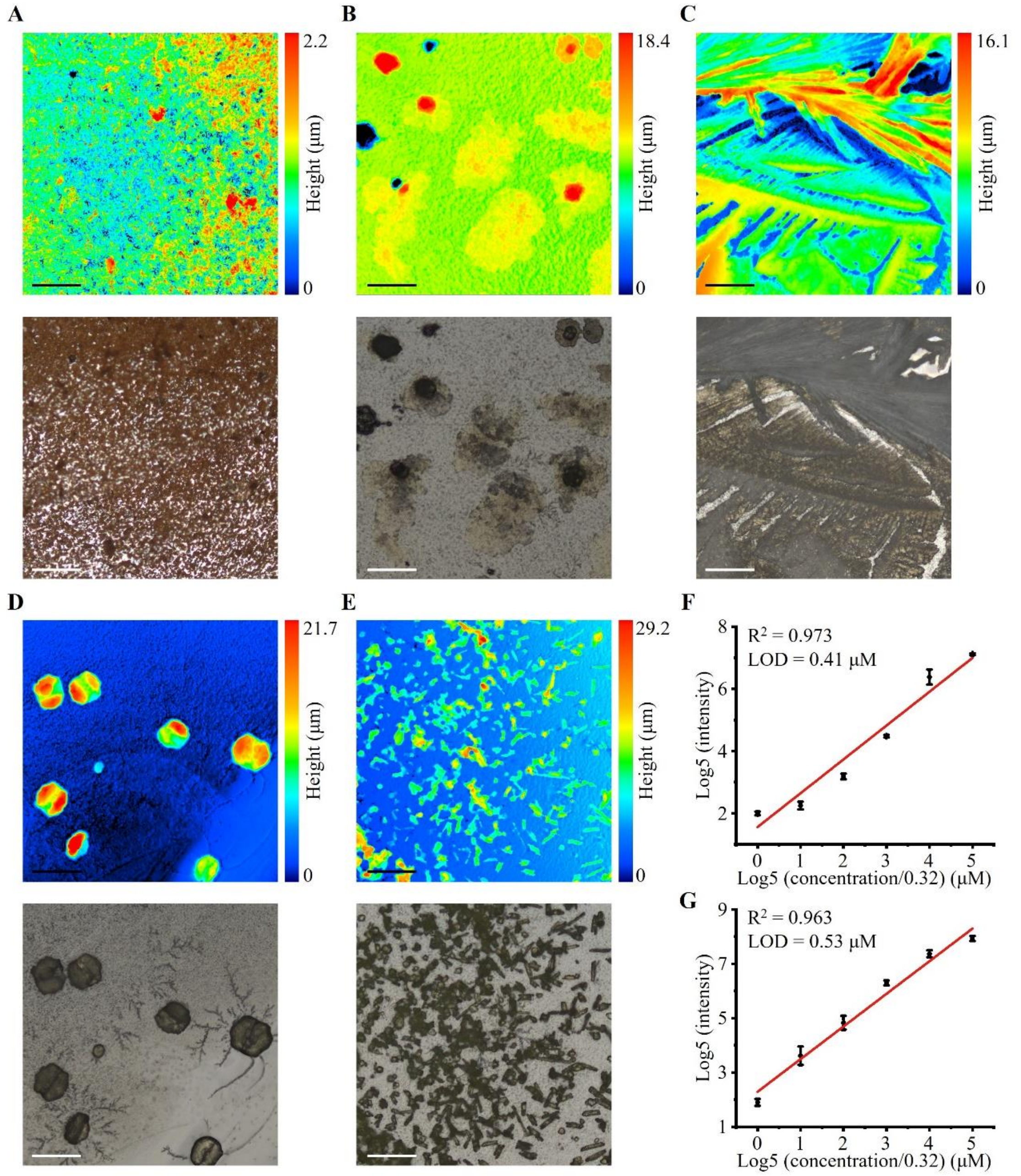

Appendix Figure S3. High reproducibility and good linear response of PELDI-MS.

A-E. 3D confocal reconstruction images displayed the homogeneous co-crystallization of **(A)** ferric oxide particles and heterogeneous co-crystallization of **(B)** CHCA, **(C)** DHB, **(D)** SA, and **(E)** DHAP. The scale bars were 100 μm.

F, G. The PELDI-MS offered a good linear response ($R^2 = 0.963\text{-}0.973$) with a limit of detection (LOD) of 0.41-0.53 μM in **(F)** alanine and **(G)** glucose analysis. Data were mean ± SD, $N = 3$ technical replicates.

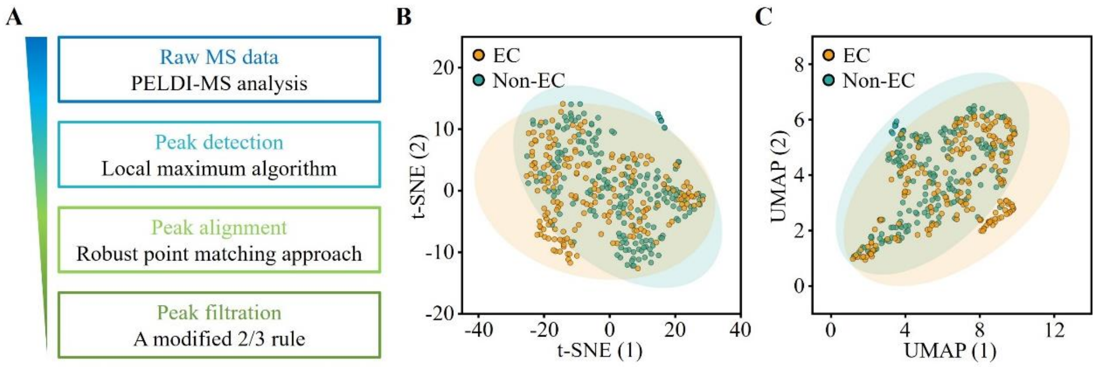

Appendix Figure S4. Data processing and SMFs Characterization of EC and Non-EC.

A. Data processing of native m/z signals, including peak detection using a local maximum algorithm, peak alignment using a robust point matching approach, and peak filtration employing a modified 2/3 rule.

B, C. The unsupervised analysis of **(B)** t-distributed stochastic neighbor embedding (t-SNE) and **(C)** uniform manifold approximation and projection (UMAP) of SMFs showed a certain degree of overlap between EC and Non-EC groups.

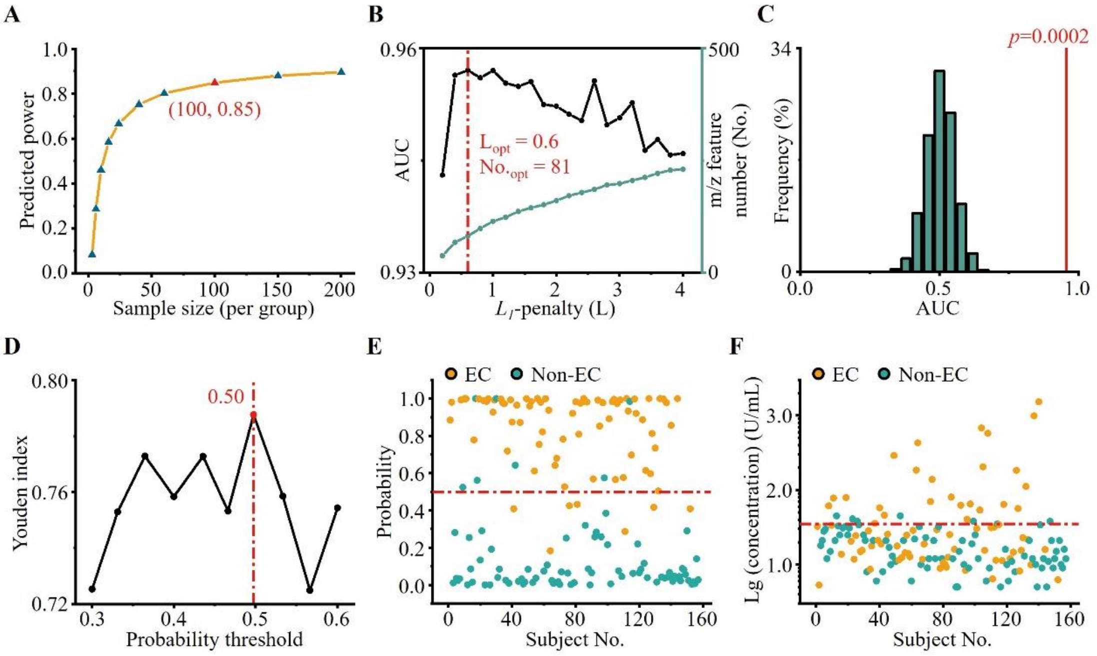

Appendix Figure S5. Machine learning of SMFs for EC diagnosis.

A. SMFs of 12 samples (6 EC and 6 Non-EC) were included in a preliminary study to determine the sample number required for statistically significant machine learning.

B. Optimization of the L_1-penalty for LASSO model in the discovery cohort. The optimized L_1-penalty (L_{opt} = 0.6) and m/z feature number ($No._{opt}$ = 81) were marked with a red dashed line.

C. The permutation test with 5000 randoms confirmed no overfitting of LASSO model.

D. Optimization of the probability threshold for SMFs with LASSO model using Youden index, and the maximum Youden index was obtained at the optimal probability threshold of 0.50.

E. Probability generated by machine learning for EC diagnosis, using LASSO as an example, for subjects (EC in orange dots, Non-EC in cyan dots) in the independent validation cohort.

F. The concentration of CA-125 for subjects (EC in orange dots, Non-EC in cyan dots) tested in clinics in the independent validation cohort.

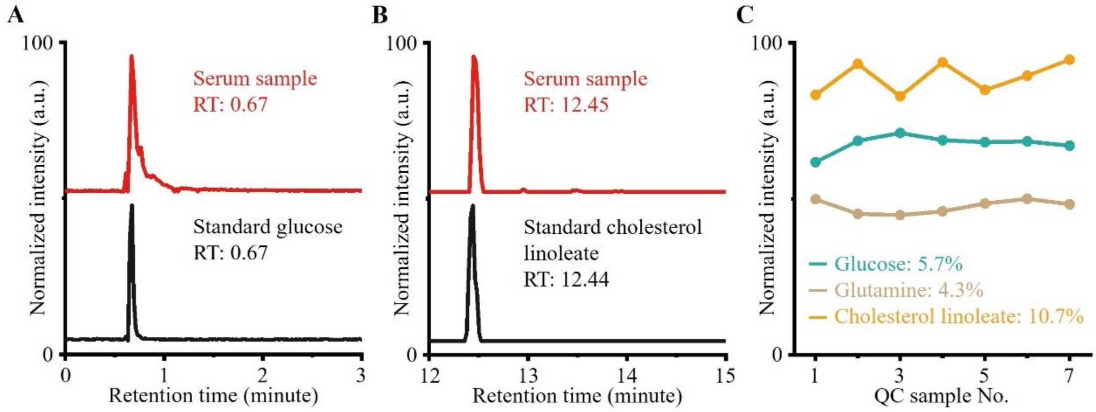

Appendix Figure S6. Metabolic biomarker panel validation in UPLC-MS.

A, B. Chromatograms of standard **(A)** glucose and **(B)** cholesterol linoleate with serum samples confirmed the reliability of metabolite detection in UPLC-MS validation.

C. Intensities of metabolites (glutamine, glucose, and cholesterol linoleate) in 7 QC samples, showing 4.3-10.7% CVs during detection in UPLC-MS validation.

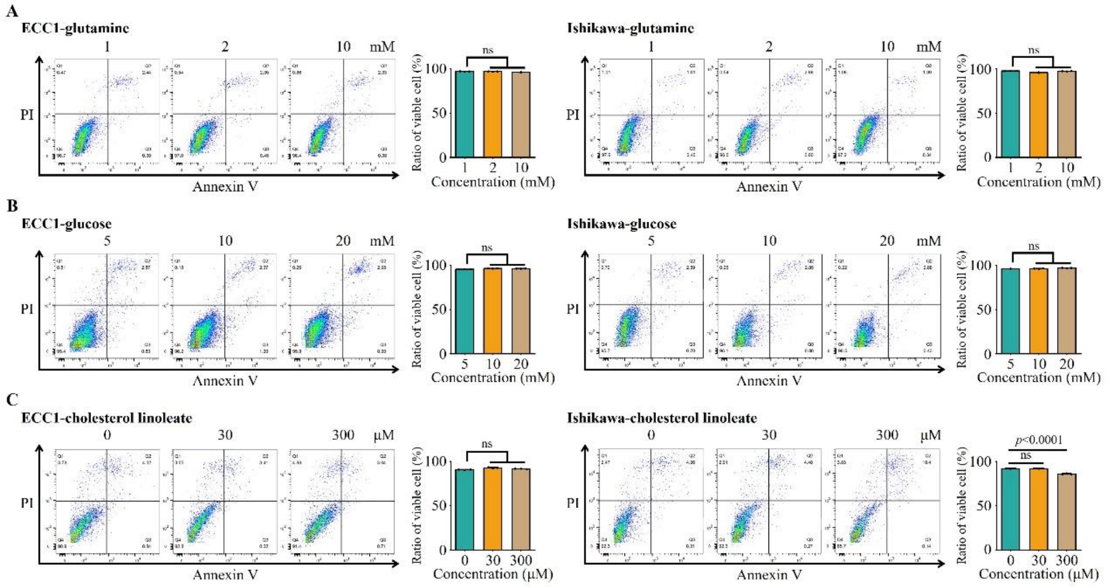

Appendix Figure S7. Apoptotic impacts of biomarkers on EC cell lines.

A, B. (A) Glutamine and **(B)** glucose did not affect the apoptosis process in EC cell lines.

C. Cholesterol linoleate induced apoptosis in Ishikawa cells at 300 μM, while demonstrating no influence on ECC1 cells. Cell apoptosis was examined by flow cytometric analysis. Data were mean ± SD, $N = 3$ biological replicates, one-way ANOVA, ns (no significance, $p > 0.05$).

Appendix Table S1. Peak intensities of metabolites for the standard sample under high salt (20 mM Na^+) and biofluid-mimicking conditions (20 mM Na^+ and 10 mg/mL protein) using different matrices (particles (Par), CHCA, DHB, SA, and DHAP).

Condition	Matrix	Alanine	Proline	Glutamic acid	Glucose	Lactose	Sum [a)]
20 mM of Na^+	Par	28494	95042	58029	63222	198034	442821
	CHCA	10	3892	1080	27	717	5726
	DHB	4	643	144	9	19	819
	SA	21	94	46	248	46	457
	DHAP	37	5768	491	25	1253	7575
20 mM of Na^+ and 10 mg/mL of protein	Par	49196	110063	50314	59035	140708	409316
	CHCA	147	515	243	230	750	1886
	DHB	30	5492	728	387	3315	9952
	SA	27	72	45	30	28	201
	DHAP	76	4459	623	25	2469	7652

a) The sum of peak intensities for alanine, proline, glutamic acid, glucose, and lactose in the standard sample under high salt (20 mM Na^+) and biofluid-mimicking conditions (20 mM Na^+ and 10 mg/mL protein) using different matrice.

Appendix Table S2. Coefficient of variation of m/z feature intensity for metabolites in the standard metabolite mixture.

Metabolite	Adduct	m/z	S.D. a)	Mean a)	CV a)
Alanine	$[M+Na]^+$	112.04	3295.69	49476.60	6.7%
	$[M-H+2Na]^+$	134.02	7328.40	99288.59	7.4%
Proline	$[M+Na]^+$	138.05	14092.60	230216.68	6.1%
	$[M-H+2Na]^+$	160.03	12120.18	150915.40	8.0%
Glutamic acid	$[M+Na]^+$	170.04	6357.95	65004.86	9.8%
	$[M-H+2Na]^+$	192.02	11973.21	114683.39	10.4%
	$[M-2H+3Na]^+$	214.01	8177.93	74248.67	11.0%
Glucose	$[M+Na]^+$	203.05	12607.42	196665.75	6.4%
Lactose	$[M+Na]^+$	365.11	19907.40	358524.07	5.6%

a) The standard deviation (S.D.), mean, and coeffitient of variance (CV) were calculated based on 16 independent replicates.

Appendix Table S3. Clinical characteristics of the enrolled EC and Non-EC subjects.

Characteristic	EC [a)]	Non-EC [a)]	*p* value
**Sample number**	191	204	–
**Age (year)**			
Mean (range)	58 (27-81)	43 (17-79)	< 0.001
**CA-125** [b)]			
Mean (range)	137 (4-3201)	20 (2-82)	< 0.001
**BMI**			
Mean (range)	25 (17-46)	23 (15-34)	< 0.001
**Diabetes**			
(No/Yes/Unknown)	149/37/5	188/16/0	< 0.001
**Menopause**			
(No/Yes/Unknown)	61/125/5	164/40/0	< 0.001
**Hypertension**			
(No/Yes/Unknown)	131/56/4	158/46/0	0.11
**FIGO Stage (%)** [c)]			
I	126 (66.0)	–	–
II	8 (4.2)	–	–
III	34 (17.8)	–	–
IV	13 (6.8)	–	–
Recurrence	10 (5.2)	–	–
	Endometrioid 169 (88.5)	Polyp/Polypoid 151 (74.0)	–
**Histology (%)**	Serous 19 (9.9)	Hyperplasia 42 (20.6)	–
	Clear cell 3 (1.6)	Adhesions 11 (5.4)	–

a) The EC and Non-EC subjects were diagnosed by two experienced pathologists independently, based on pathological examination.

b) The concentration of CA-125 (U/mL) for EC and Non-EC subjects was assessed in the clinical setting.

c) The stage information of EC was determined according to the FIGO 2018 standard.

Appendix Table S4. Performance of the 5 machine learning algorithms for EC diagnosis in the discovery cohort.

**Algorithm**	**AUC [a)]**	**95% CI [a)]**	***p* value**
LASSO	0.957	0.906-1.000	–
Logistic regression	0.940	0.876-0.998	< 0.001
PLS-DA	0.940	0.877-0.996	< 0.001
Random forest	0.905	0.822-0.986	< 0.001
Decision tree	0.757	0.635-0.879	< 0.001

a) The area-under-the-curve (AUC) and corresponding 95% confidence interval (CI) were calculated in the discovery cohort.

Appendix Table S5. Sensitivity and specificity of the SMFs and CA-125 for EC diagnosis and early-stage (FIGO 2018 stage I/II) EC diagnosis in both the discovery and validation cohorts.

	**Diagnostic approach** [a)]	**Cohort**	**Sensitivity**	**Specificity**
**Diagnosis**	SMFs	Discovery	86.1%	91.9%
		Validation	90.8%	91.4%
	CA-125	Discovery	37.4%	74.8%
		Validation	32.9%	91.4%
**Early diagnosis**	SMFs	Discovery	85.7%	91.9%
		Validation	91.2%	91.4%
	CA-125	Discovery	22.1%	74.8%
		Validation	26.3%	91.4%

a) The sensitivity and specificity were calculated based on the SMFs (272 m/z features) with LASSO model or the concentration of CA-125 in serum.

Appendix Table S6. The information of identified biomarkers for the differentiation of EC and Non-EC.

**Measured m/z** [a)]	**Error (ppm)** [b)]	**Molecular formula** [c)]	**Metabolite** [d)]	**Adduct** [c)]	**HMDB ID** [d)]
212.957	\	\	\	\	\
203.052	-1.37	$C_6H_{12}O_6$	Glucose	$[M+Na]^+$	HMDB0000122
204.056*	-1.52			$[M+Na]^+$	
219.026	-2.31			$[M+K]^+$	
222.988	-0.86	$C_5H_{10}N_2O_3$	Glutamine	$[M-H+2K]^+$	HMDB0000641
671.575	2.59	$C_{45}H_{76}O_2$	Cholesterol linoleate	$[M+Na]^+$	HMDB0000610
672.579*	2.69			$[M+Na]^+$	

a) The accurate m/z value was measured using the FT-ICR-MS with high resolution, and the * referred to the relevant ions of isotopes.

b) The error of measured m/z was calculated as (measured m/z - theoretical m/z) / theoretical m/z.

c) The molecular formula and adduct species were annotated based on accurate m/z measurements (< 3 ppm).

d) The metabolite name and HMDB ID were searched in HMDB based on the molecular formula.

Appendix Table S7. Odds ratio of Met-score and potentially relevant variables (Age, BMI, diabetes, and menopause).

Covariate	Odds ratio (95% CI)	Significance (*p* value)
**Met-score**	7.356 (5.924-9.143)	< 0.001
**Age**	3.357 (2.465-4.572)	< 0.001
**BMI**	1.158 (0.973-1.379)	0.397
**Diabetes**	0.394 (0.240-0.645)	0.059
**Menopause**	1.241 (0.752-2.048)	0.666
