# Supplementary figures and images for: Identification and validation of serum metabolite biomarkers for endometrial cancer diagnosis
Source: EMBO Mol Med. 2024 Feb 14;16(4):988–1003. doi: 10.1038/s44321-024-00033-1 (PMC11018850; doi:10.1038/s44321-024-00033-1)

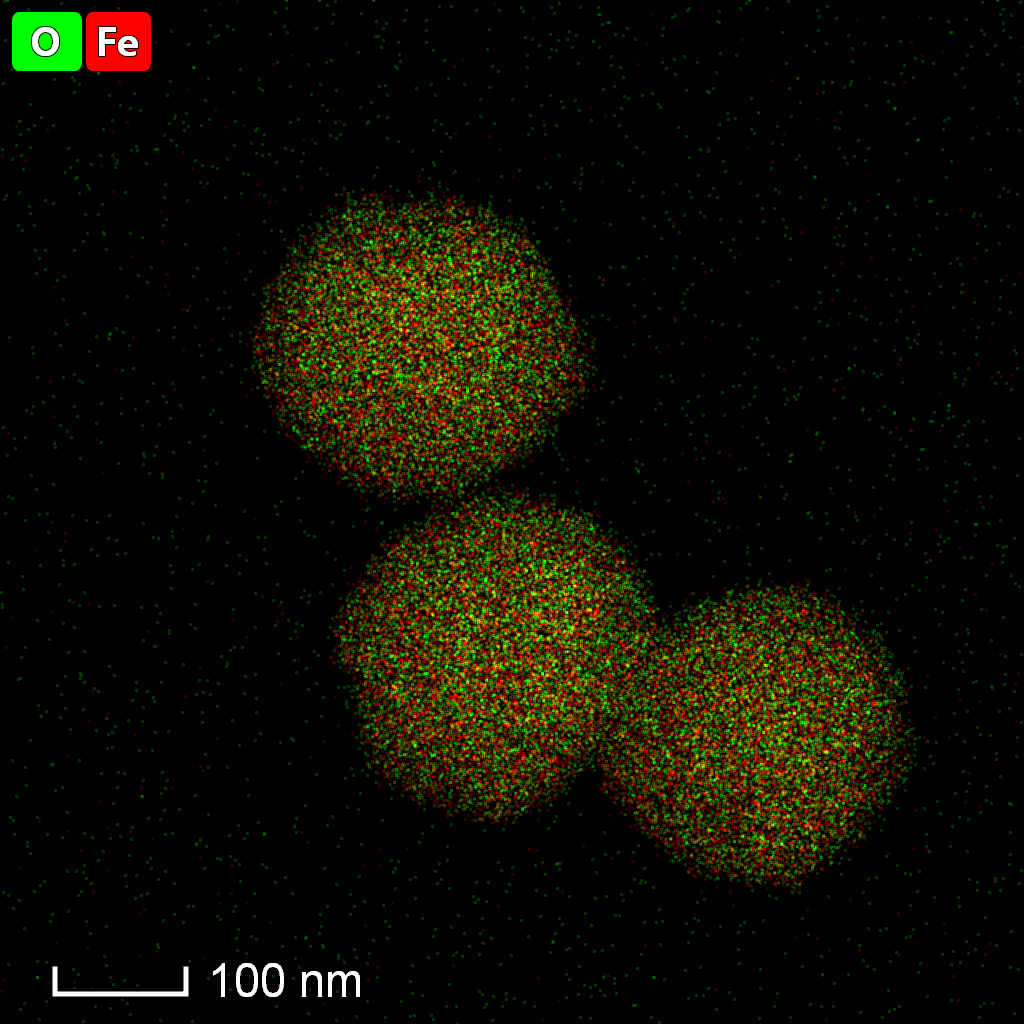

Supplement: Supplementary file 2 — Source Data Fig. 2 [file 44321_2024_33_MOESM2_ESM.zip › Figure 2/2C/Fe+O.tif]

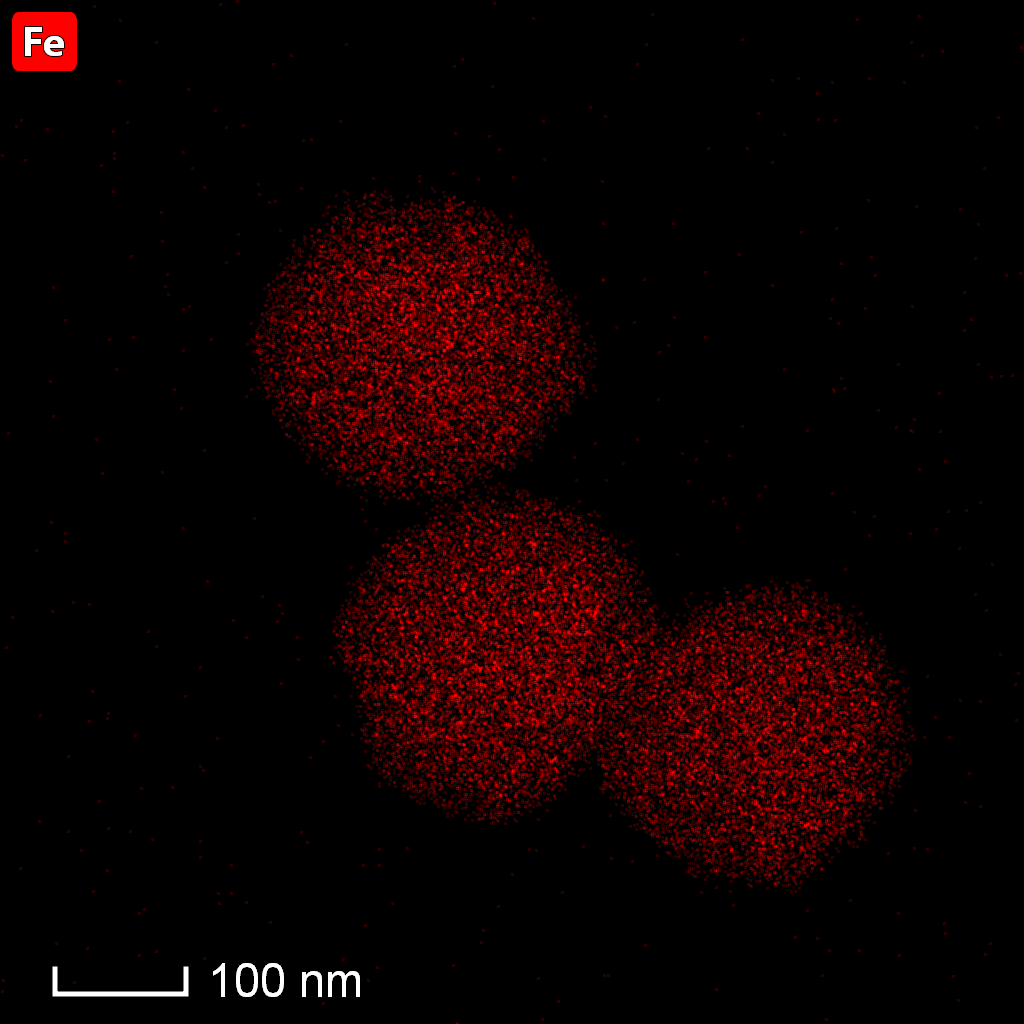

Supplement: Supplementary file 2 — Source Data Fig. 2 [file 44321_2024_33_MOESM2_ESM.zip › Figure 2/2C/Fe.tif]

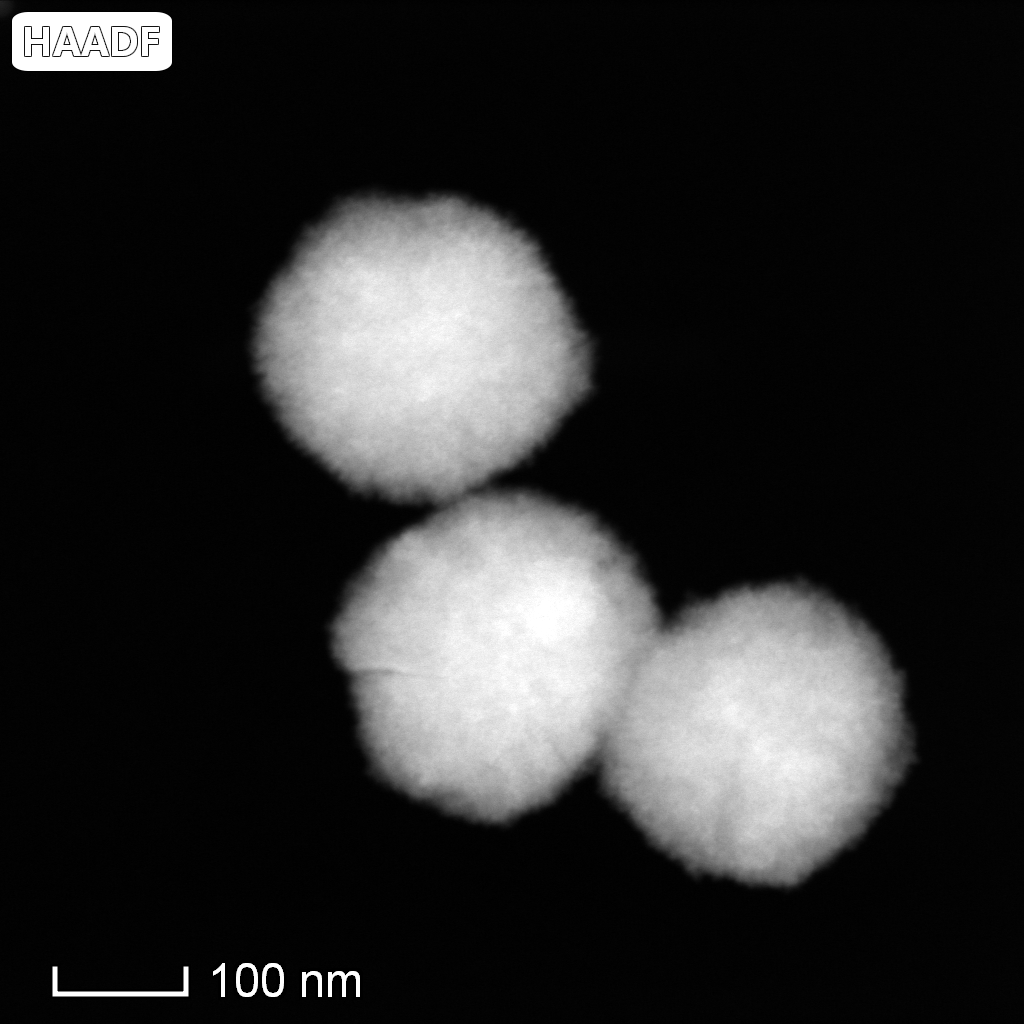

Supplement: Supplementary file 2 — Source Data Fig. 2 [file 44321_2024_33_MOESM2_ESM.zip › Figure 2/2C/HAADF.tif]

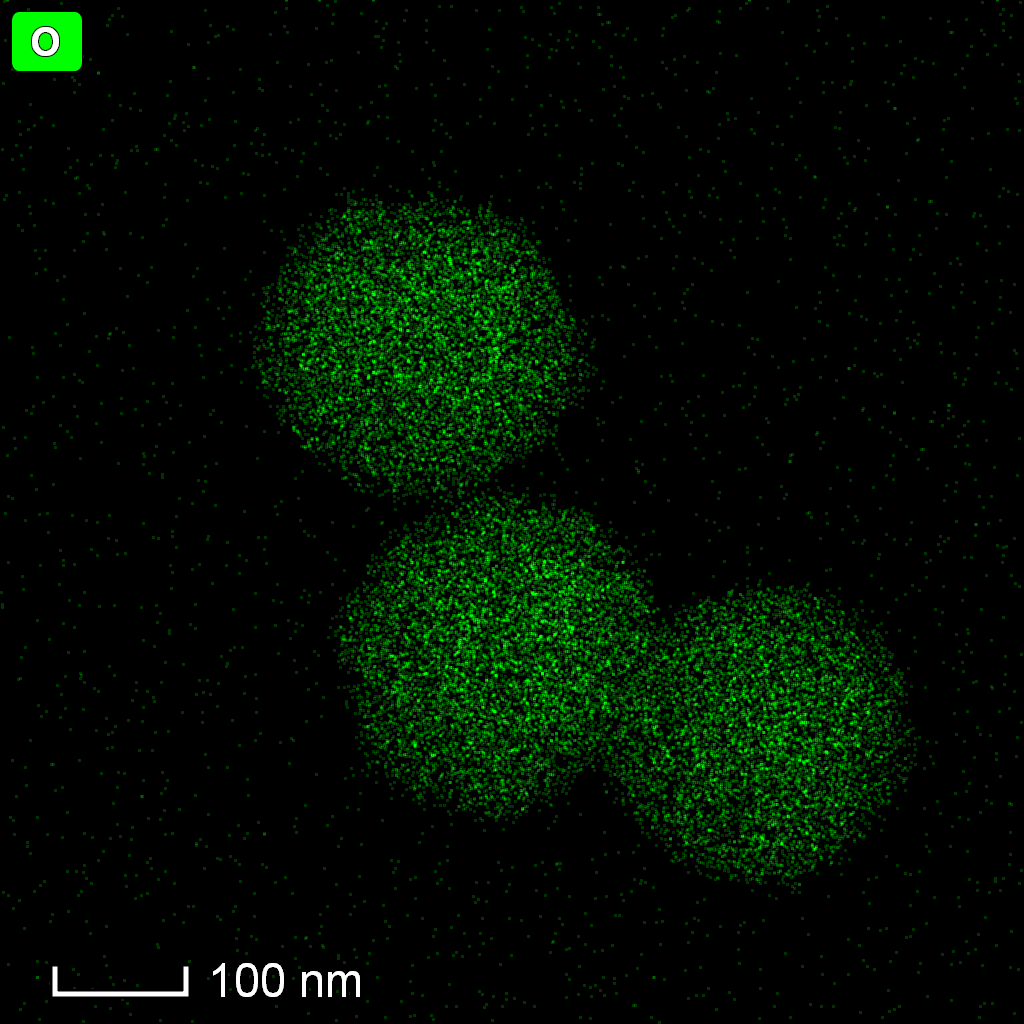

Supplement: Supplementary file 2 — Source Data Fig. 2 [file 44321_2024_33_MOESM2_ESM.zip › Figure 2/2C/O.tif]

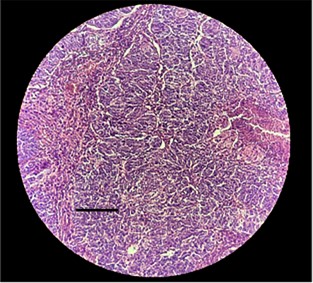

Supplement: Supplementary file 4 — Source Data Fig. 4 [file 44321_2024_33_MOESM4_ESM.zip › Figure 4/4H/Case 1-H&E staining.jpg]

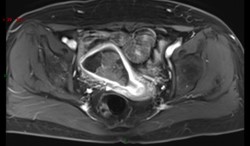

Supplement: Supplementary file 4 — Source Data Fig. 4 [file 44321_2024_33_MOESM4_ESM.zip › Figure 4/4H/Case 1-MRI.jpg]

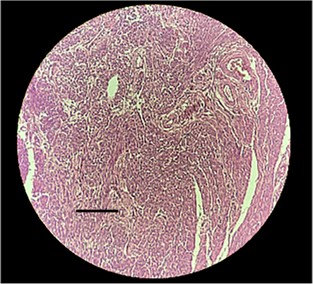

Supplement: Supplementary file 4 — Source Data Fig. 4 [file 44321_2024_33_MOESM4_ESM.zip › Figure 4/4H/Case 2-H&E staining.jpg]

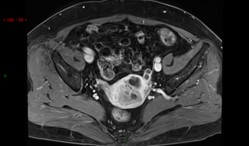

Supplement: Supplementary file 4 — Source Data Fig. 4 [file 44321_2024_33_MOESM4_ESM.zip › Figure 4/4H/Case 2-MRI.jpg]

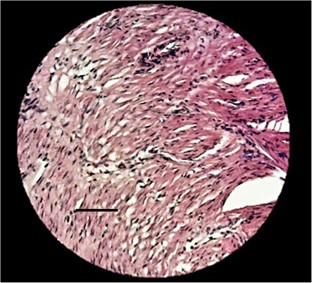

Supplement: Supplementary file 4 — Source Data Fig. 4 [file 44321_2024_33_MOESM4_ESM.zip › Figure 4/4H/Case 3-H&E staining.jpg]

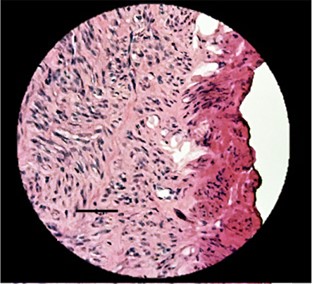

Supplement: Supplementary file 4 — Source Data Fig. 4 [file 44321_2024_33_MOESM4_ESM.zip › Figure 4/4H/Case 4-H&E staining.jpg]

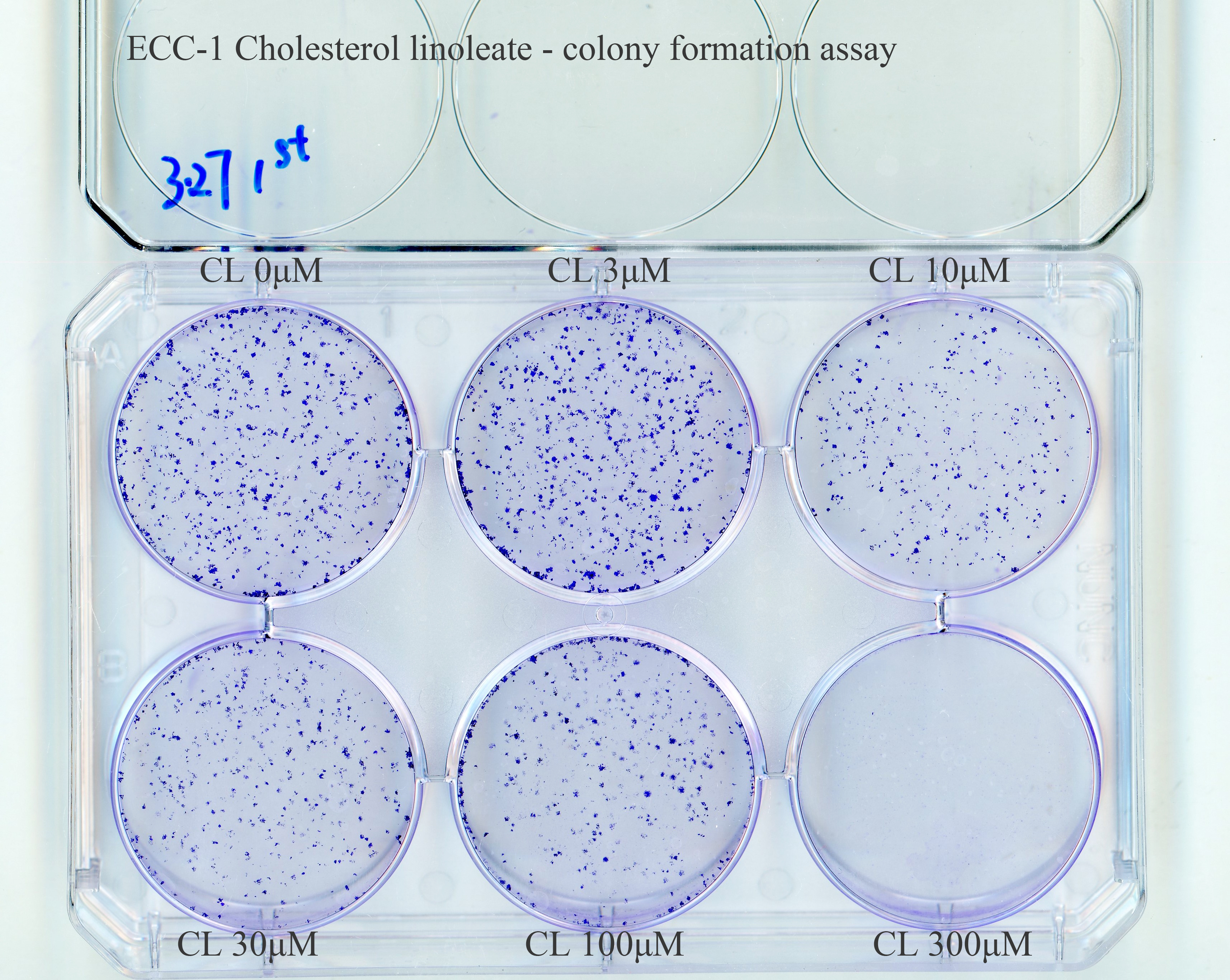

Supplement: Supplementary file 6 — Source Data Fig. 6 [file 44321_2024_33_MOESM6_ESM.zip › Figure 6/6B/ECC1 Cholesterol linoleate.jpg]

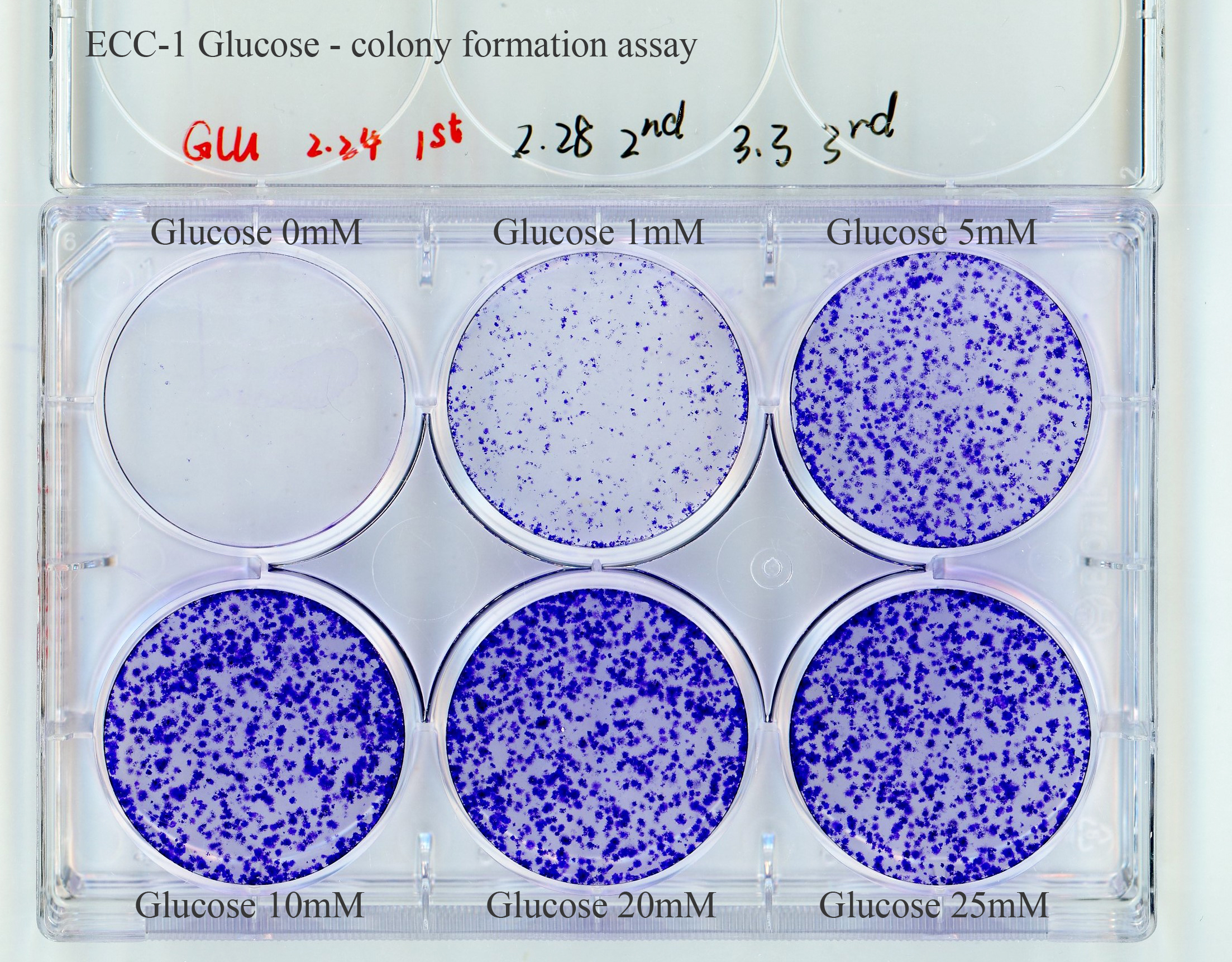

Supplement: Supplementary file 6 — Source Data Fig. 6 [file 44321_2024_33_MOESM6_ESM.zip › Figure 6/6B/ECC1 Glucose.jpg]

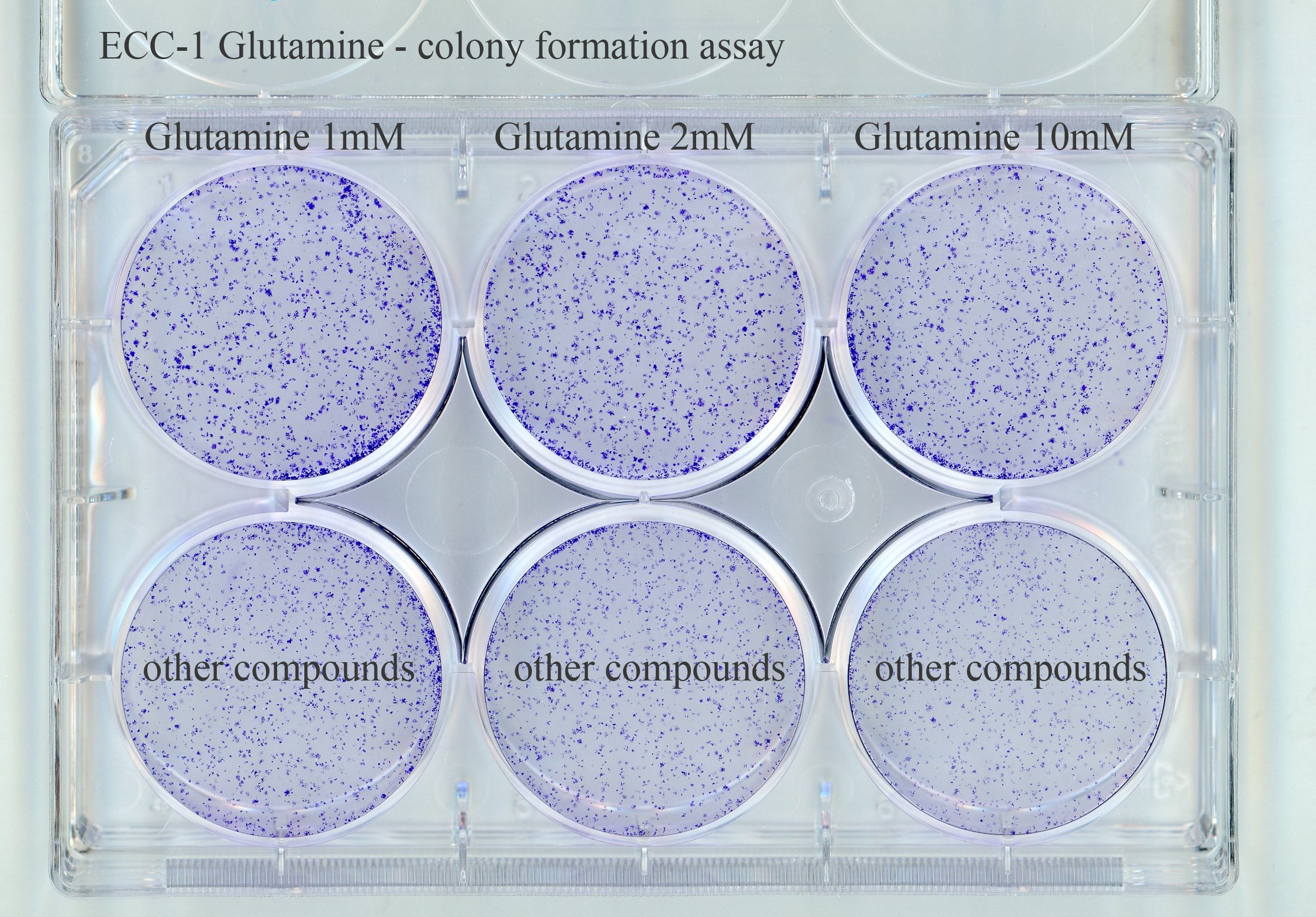

Supplement: Supplementary file 6 — Source Data Fig. 6 [file 44321_2024_33_MOESM6_ESM.zip › Figure 6/6B/ECC1 Glutamine.jpg]

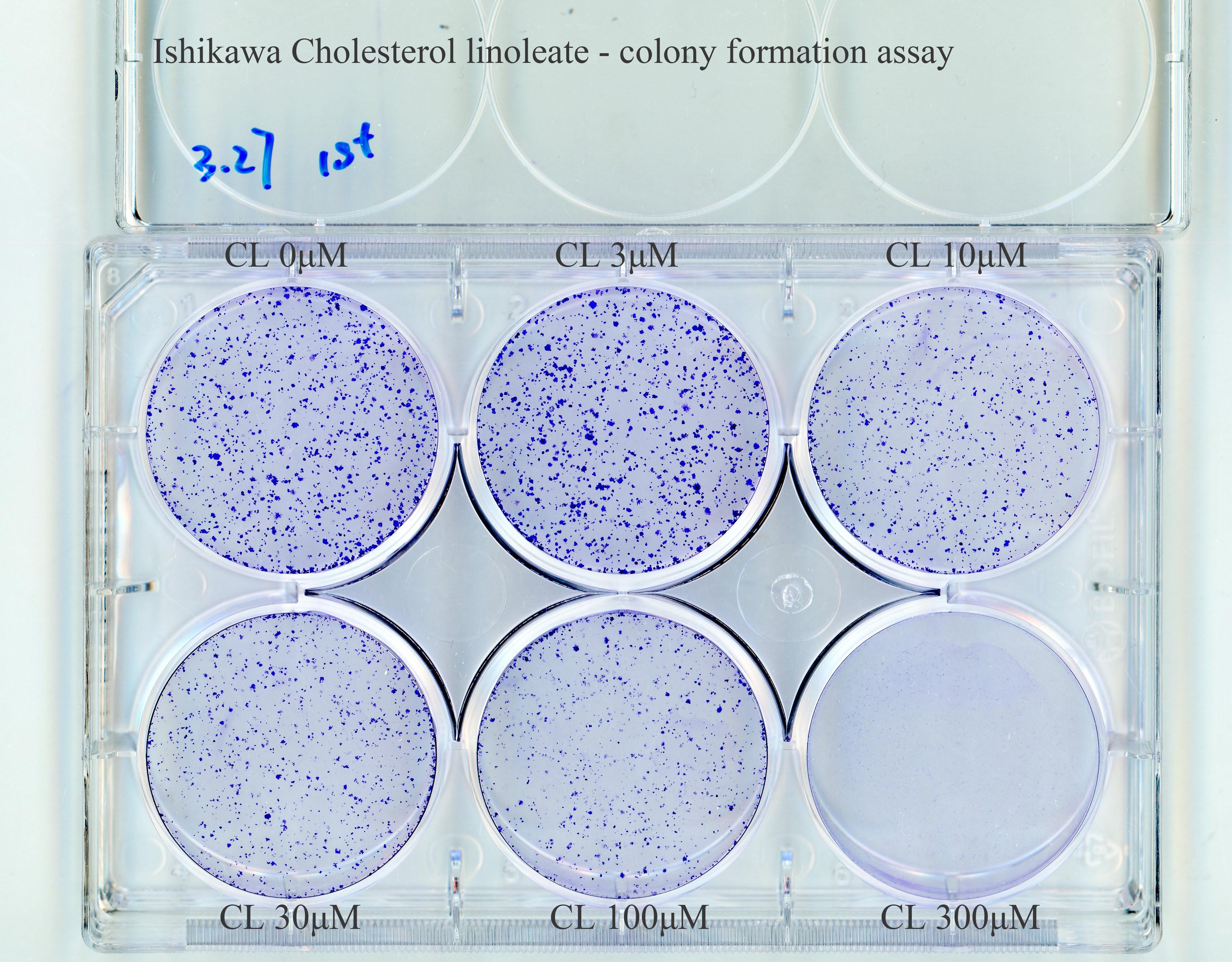

Supplement: Supplementary file 6 — Source Data Fig. 6 [file 44321_2024_33_MOESM6_ESM.zip › Figure 6/6B/Ishikawa Cholesterol linoleate.jpg]

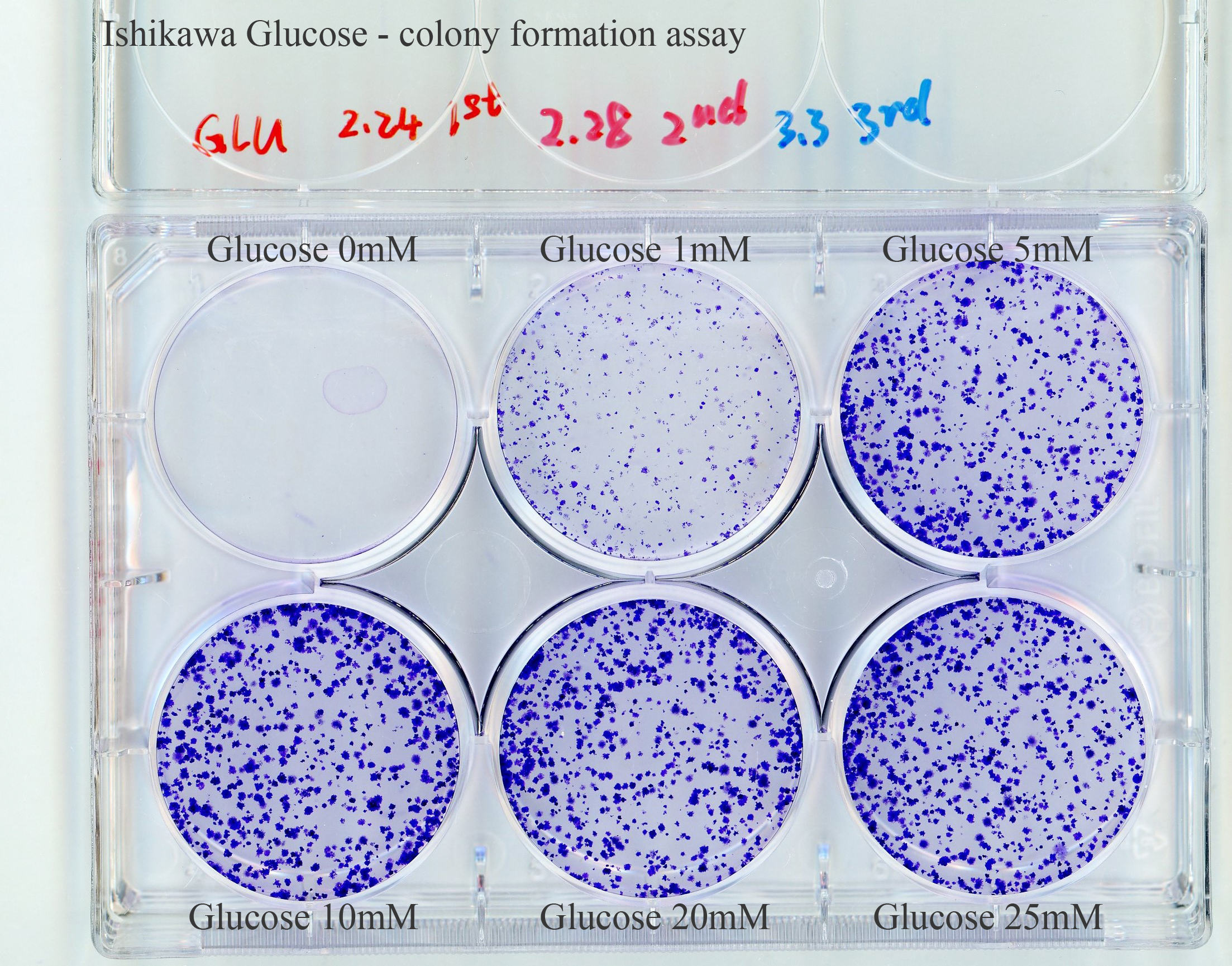

Supplement: Supplementary file 6 — Source Data Fig. 6 [file 44321_2024_33_MOESM6_ESM.zip › Figure 6/6B/Ishikawa Glucose.jpg]

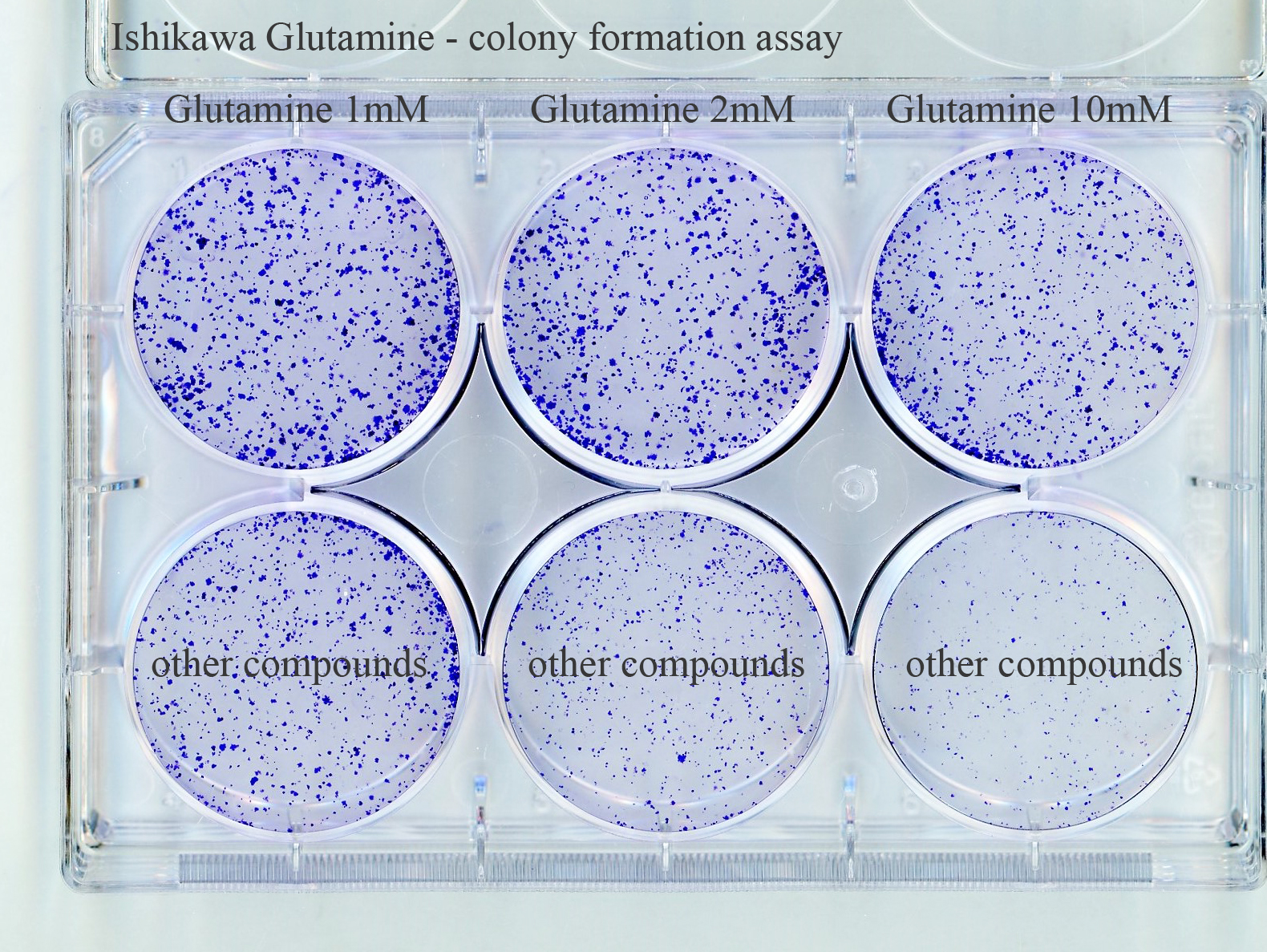

Supplement: Supplementary file 6 — Source Data Fig. 6 [file 44321_2024_33_MOESM6_ESM.zip › Figure 6/6B/Ishikawa Glutamine.jpg]

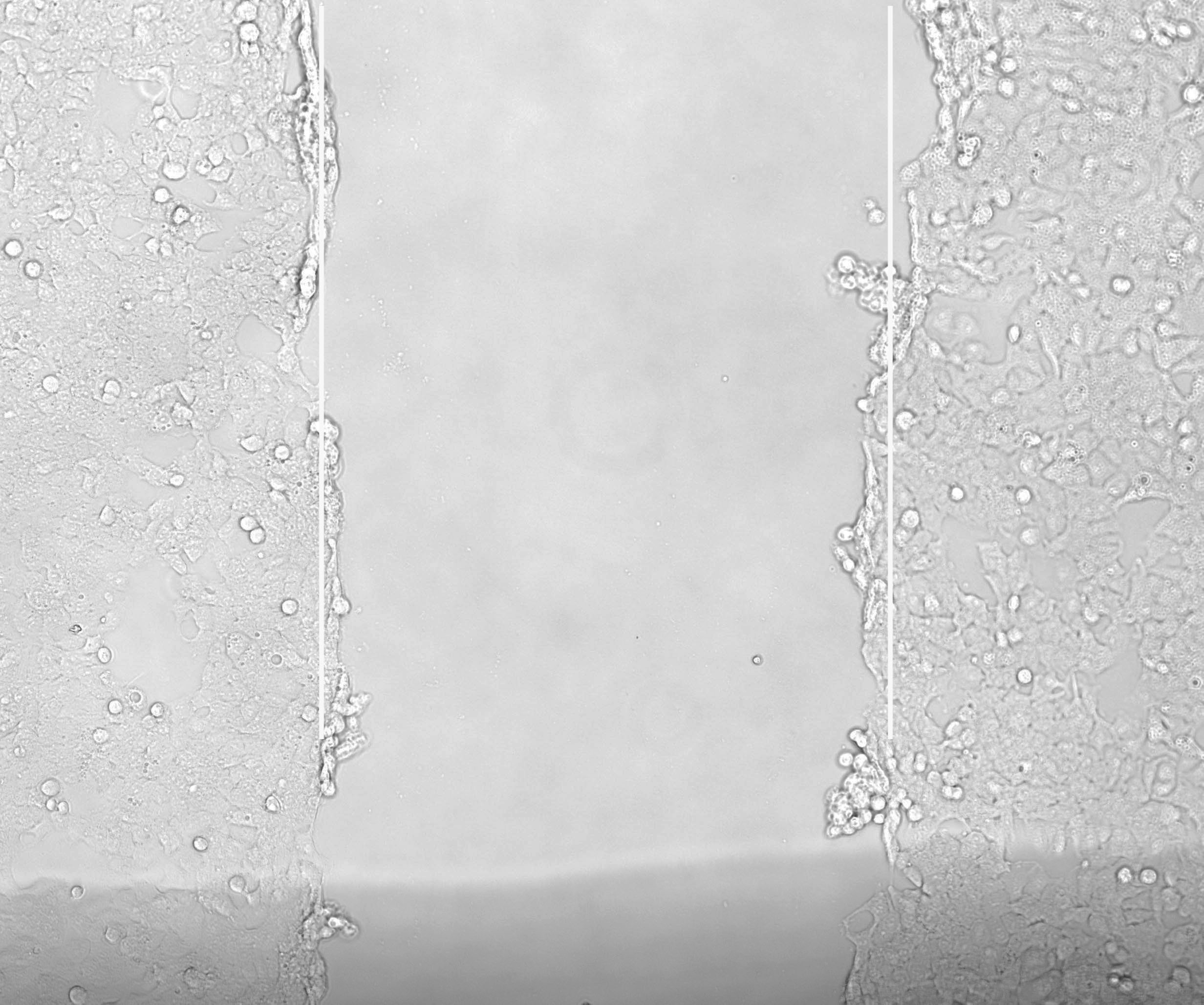

Supplement: Supplementary file 6 — Source Data Fig. 6 [file 44321_2024_33_MOESM6_ESM.zip › Figure 6/6C/ECC1 Cholesterol linoleate/0H-0μM.jpg]

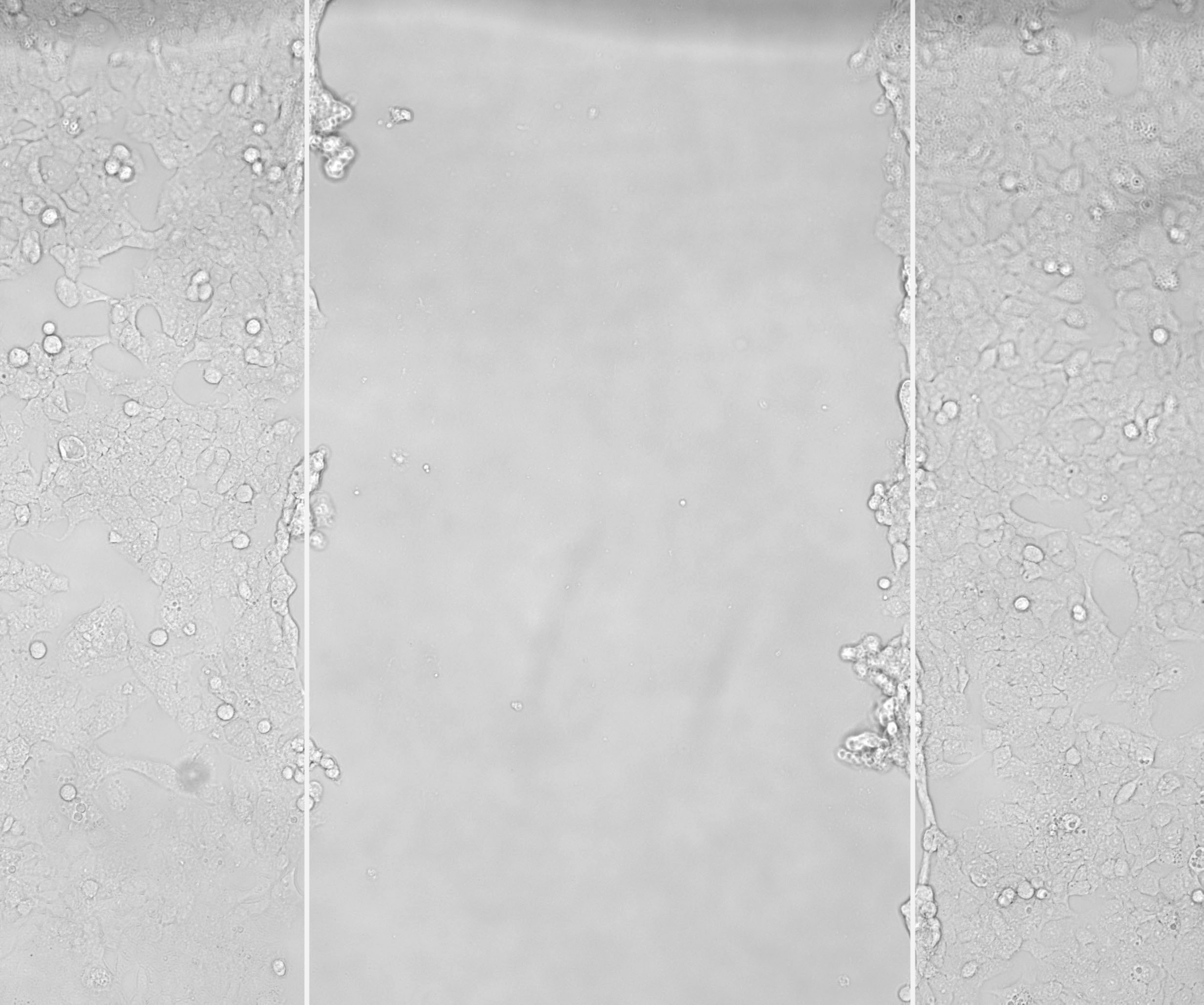

Supplement: Supplementary file 6 — Source Data Fig. 6 [file 44321_2024_33_MOESM6_ESM.zip › Figure 6/6C/ECC1 Cholesterol linoleate/0H-300μM.jpg]

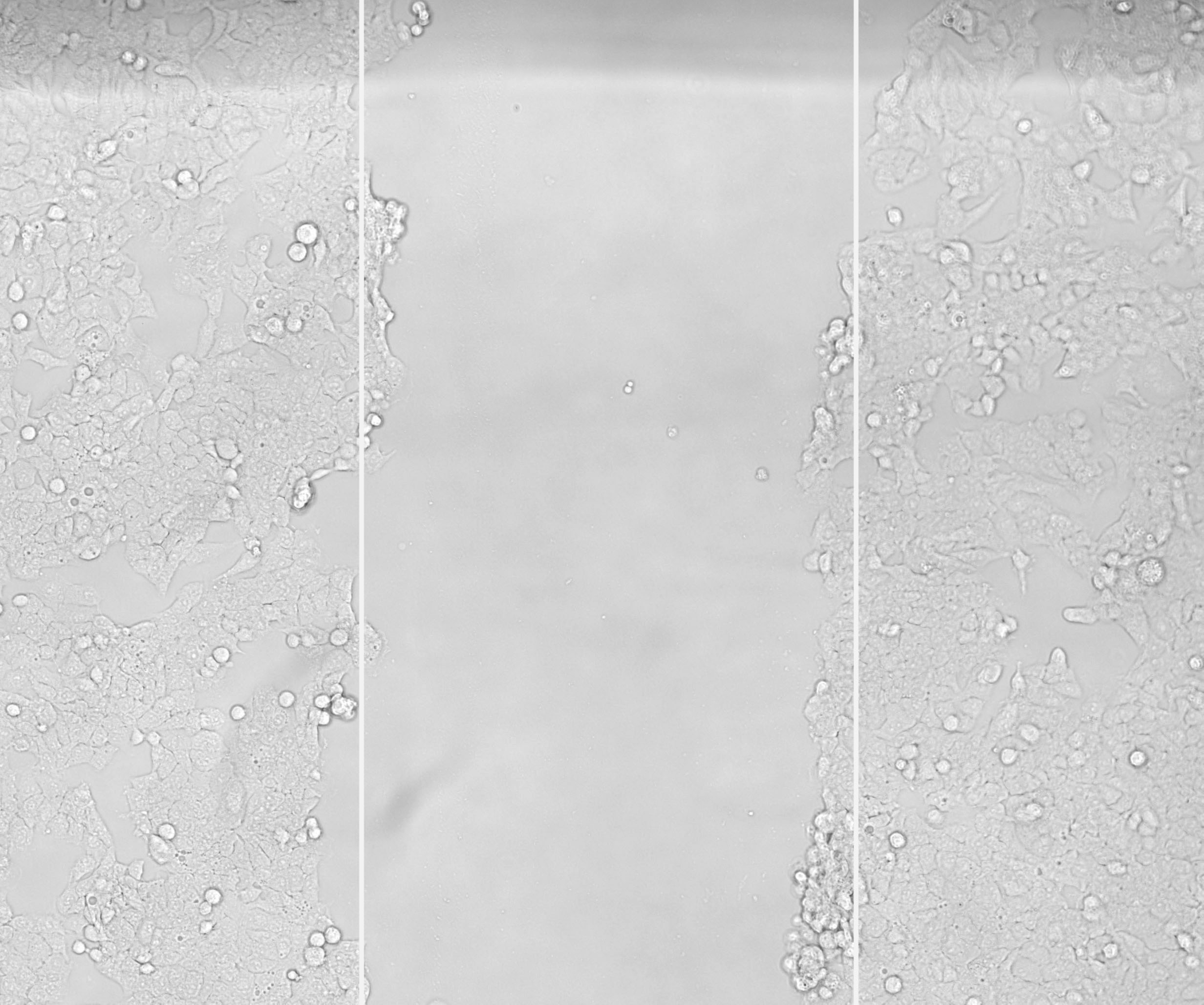

Supplement: Supplementary file 6 — Source Data Fig. 6 [file 44321_2024_33_MOESM6_ESM.zip › Figure 6/6C/ECC1 Cholesterol linoleate/0H-30μM.jpg]

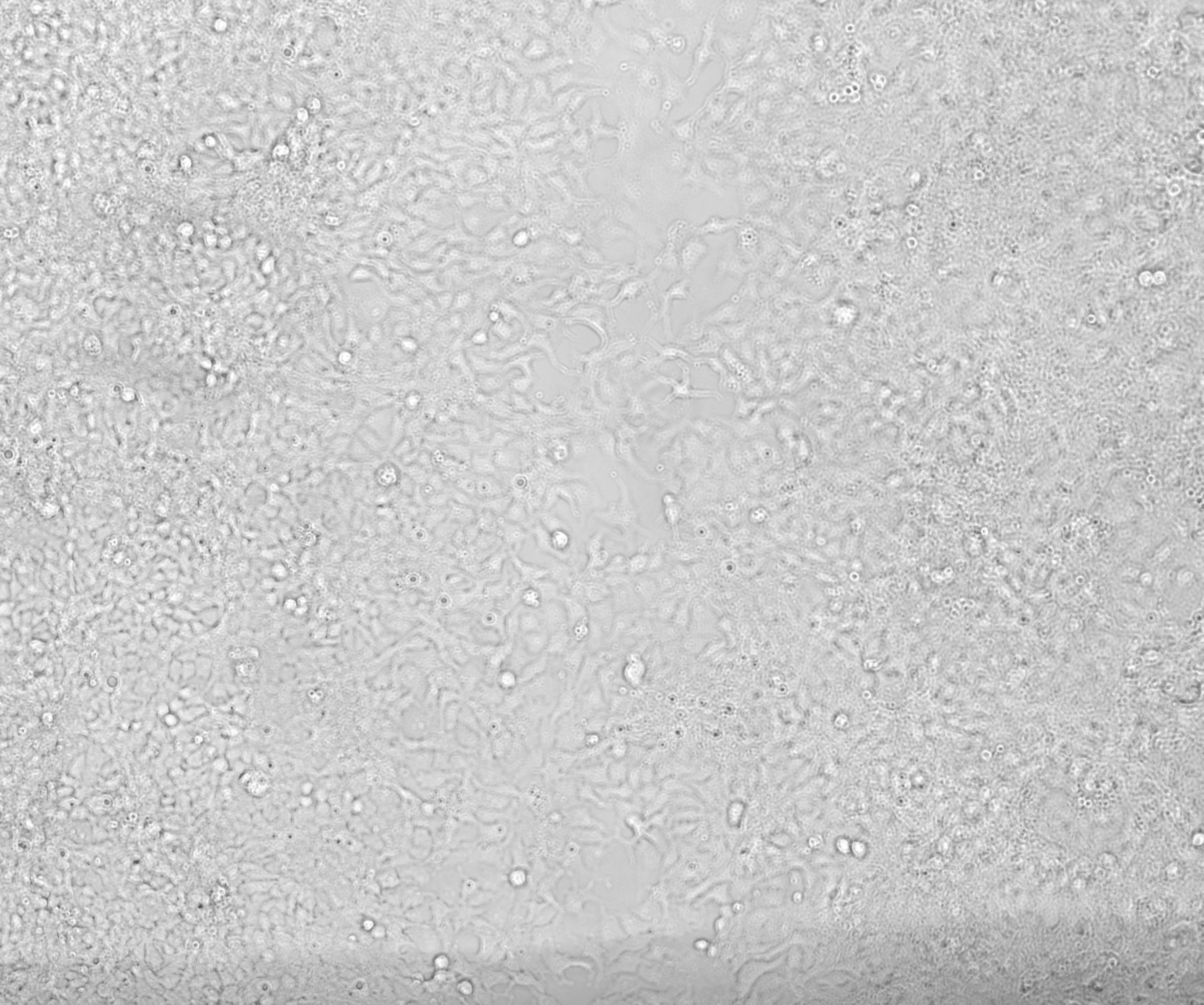

Supplement: Supplementary file 6 — Source Data Fig. 6 [file 44321_2024_33_MOESM6_ESM.zip › Figure 6/6C/ECC1 Cholesterol linoleate/48H-0μM.jpg]

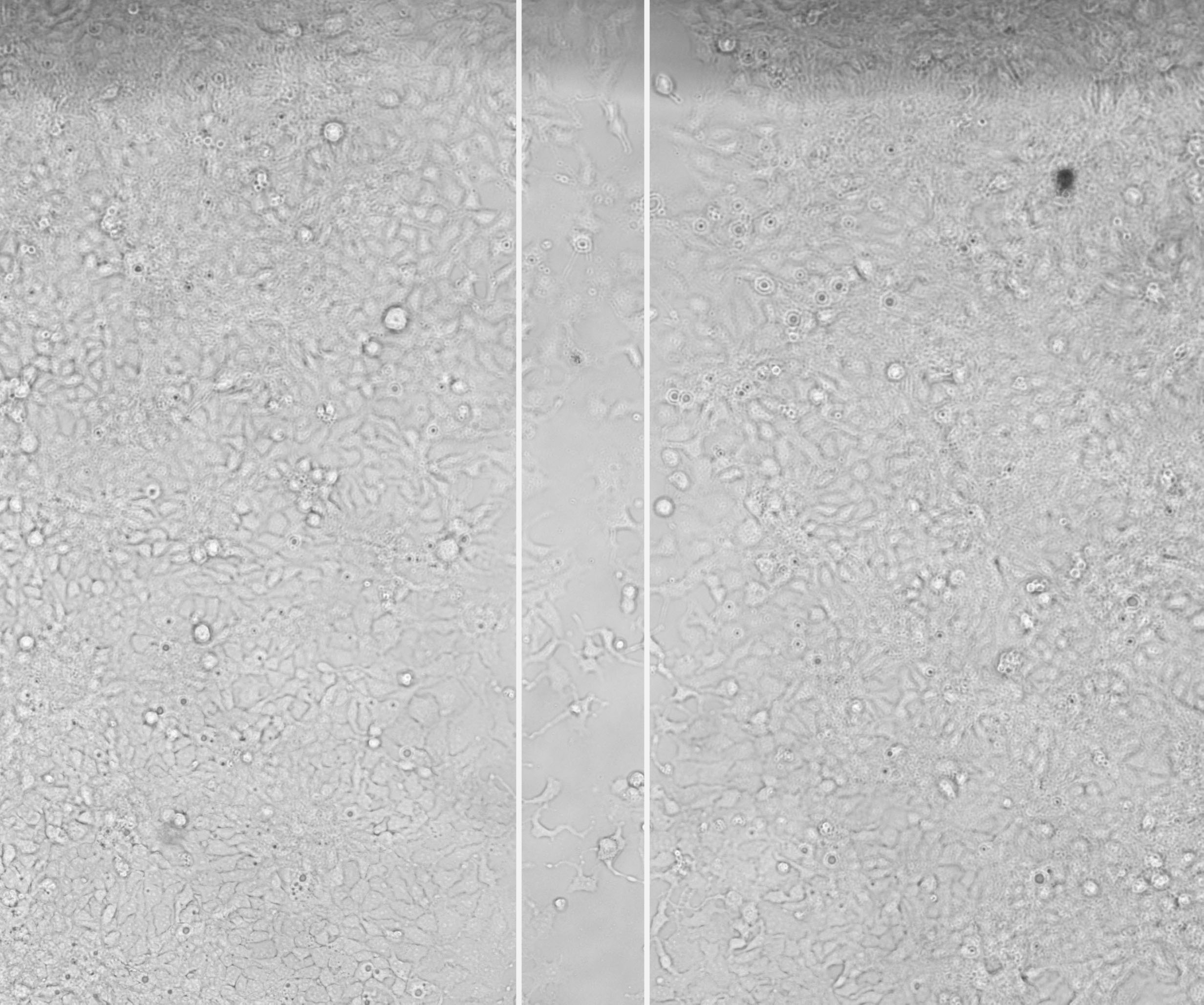

Supplement: Supplementary file 6 — Source Data Fig. 6 [file 44321_2024_33_MOESM6_ESM.zip › Figure 6/6C/ECC1 Cholesterol linoleate/48H-300μM.jpg]

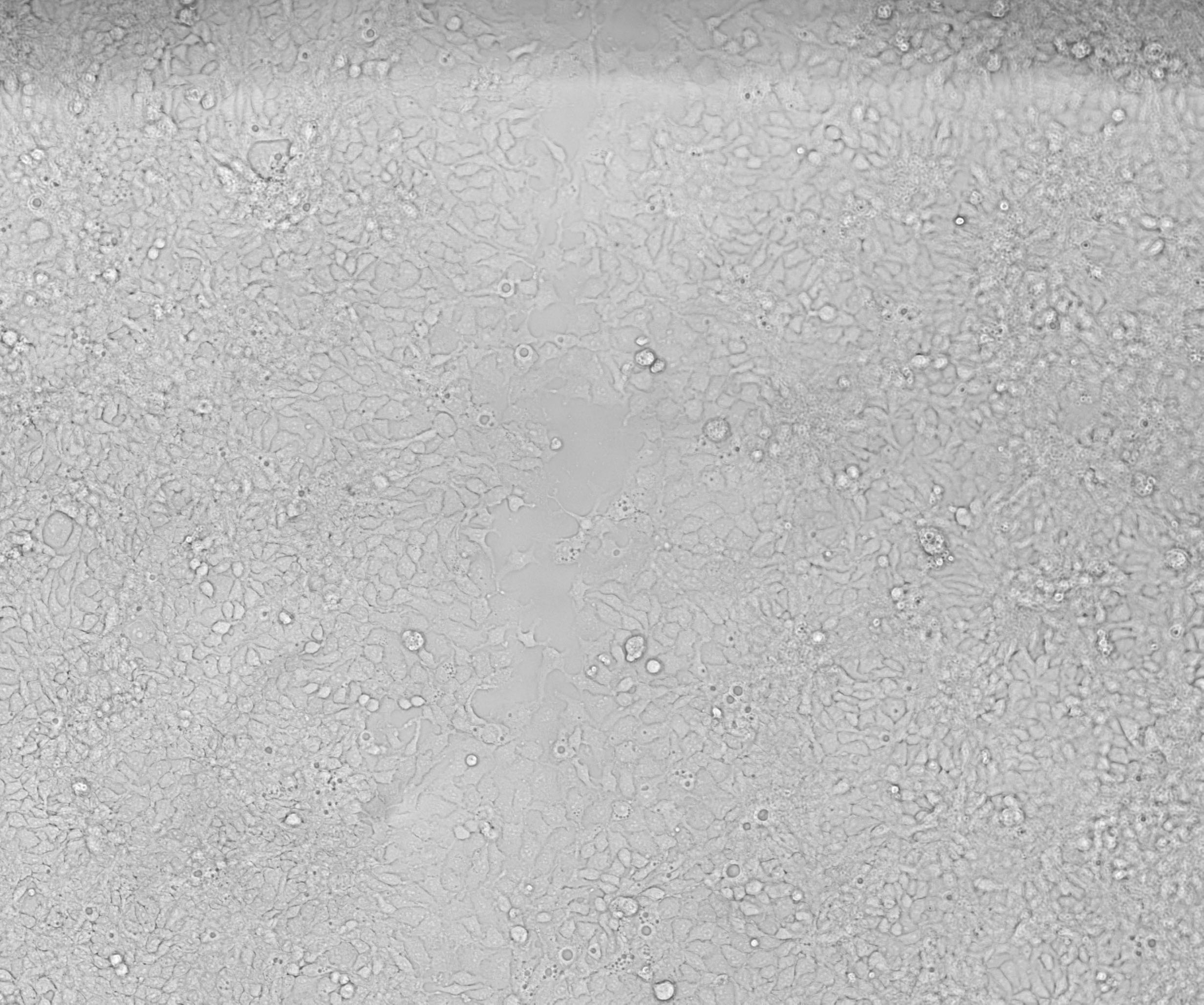

Supplement: Supplementary file 6 — Source Data Fig. 6 [file 44321_2024_33_MOESM6_ESM.zip › Figure 6/6C/ECC1 Cholesterol linoleate/48H-30μM.jpg]

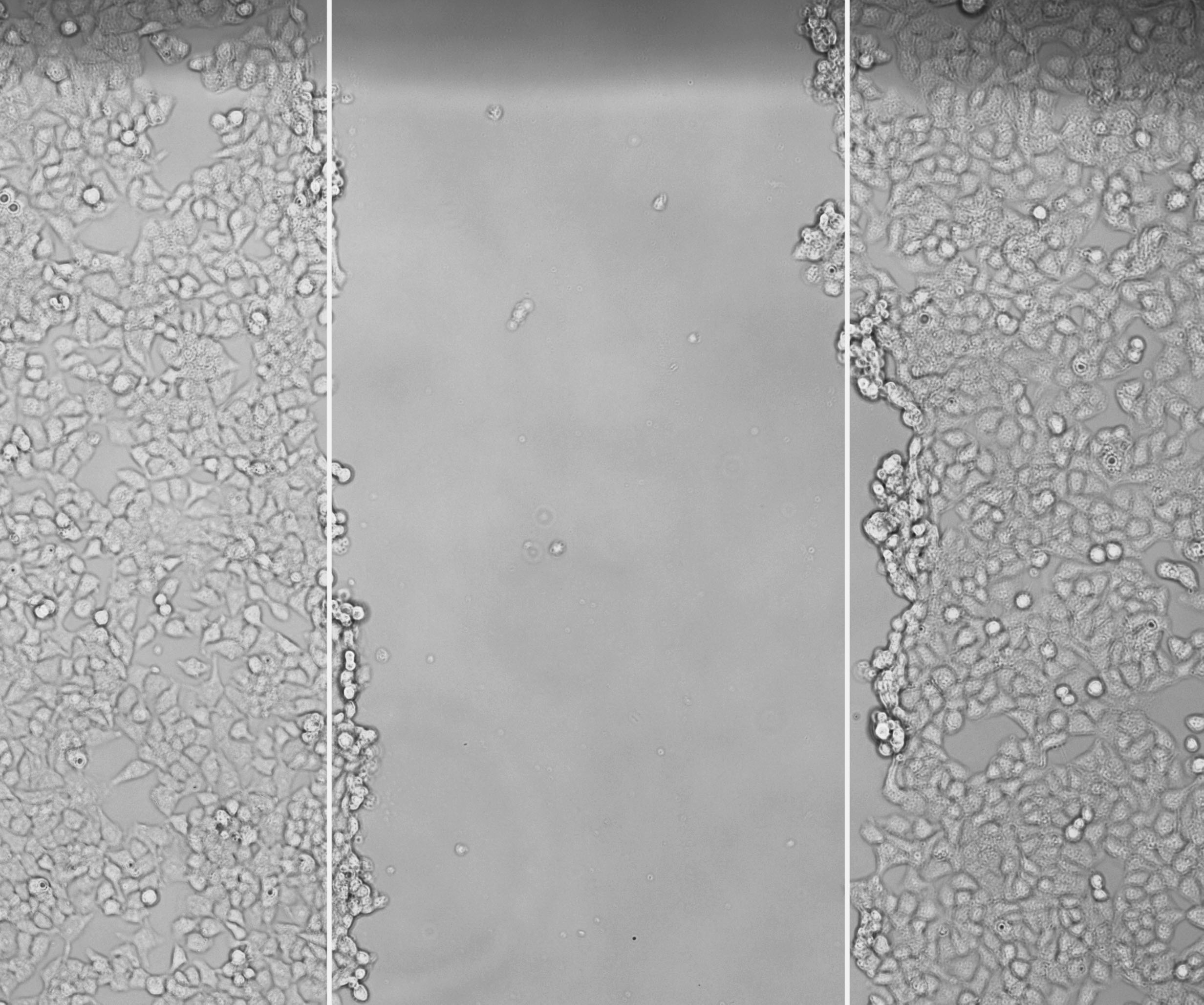

Supplement: Supplementary file 6 — Source Data Fig. 6 [file 44321_2024_33_MOESM6_ESM.zip › Figure 6/6C/ECC1 Glucose/0H-10mM.jpg]

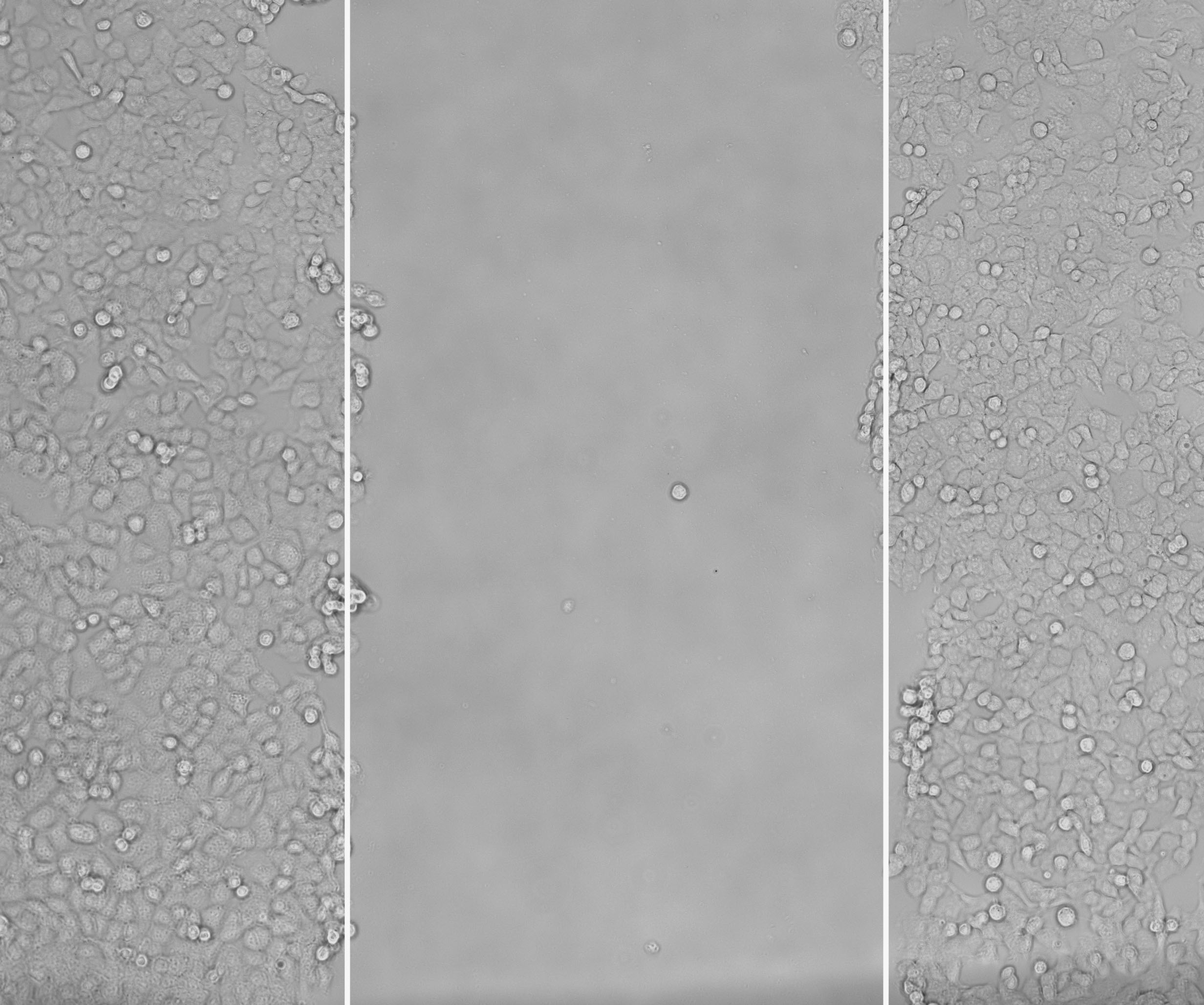

Supplement: Supplementary file 6 — Source Data Fig. 6 [file 44321_2024_33_MOESM6_ESM.zip › Figure 6/6C/ECC1 Glucose/0H-20mM.jpg]

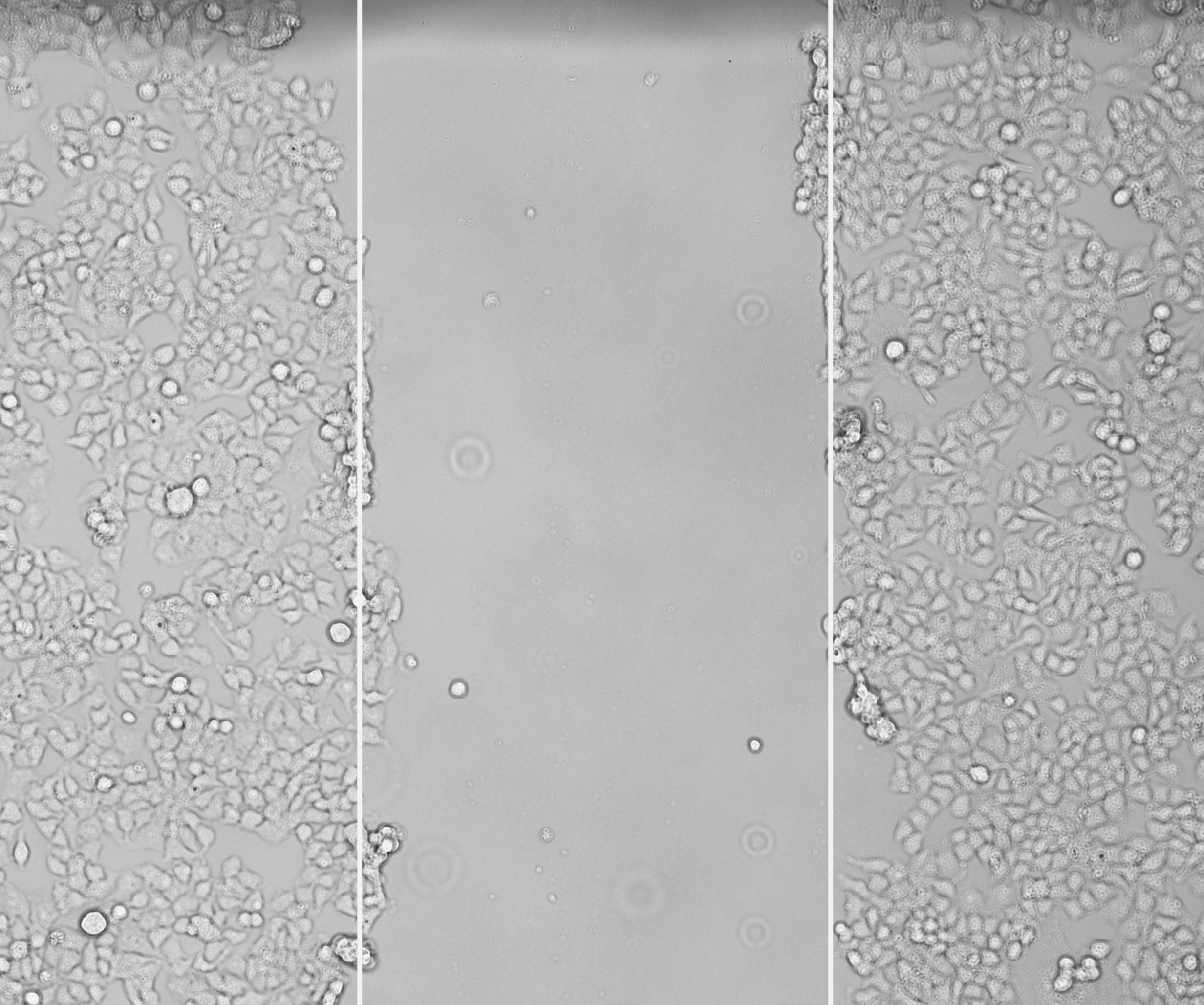

Supplement: Supplementary file 6 — Source Data Fig. 6 [file 44321_2024_33_MOESM6_ESM.zip › Figure 6/6C/ECC1 Glucose/0H-5mM.jpg]

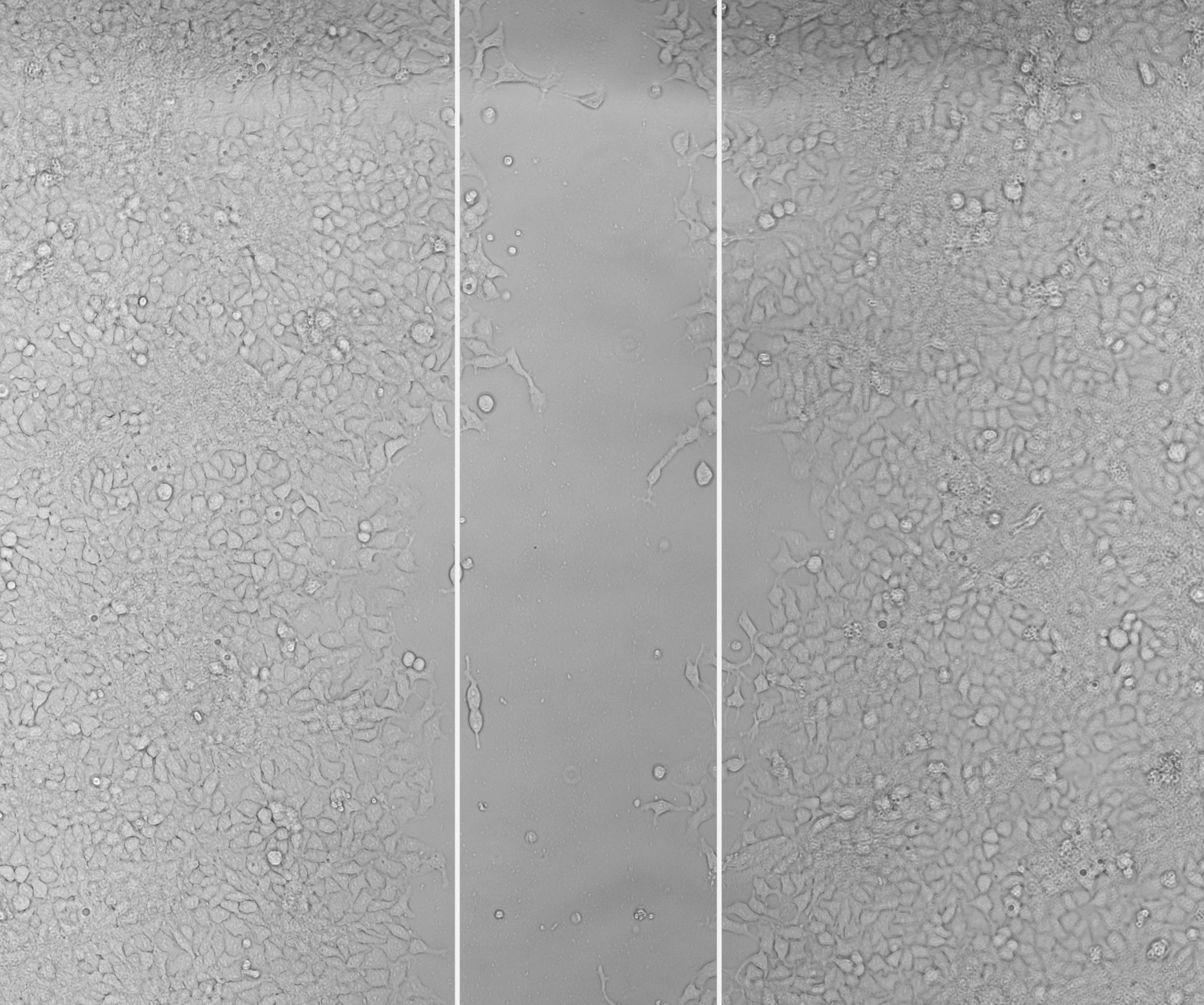

Supplement: Supplementary file 6 — Source Data Fig. 6 [file 44321_2024_33_MOESM6_ESM.zip › Figure 6/6C/ECC1 Glucose/48H-10mM.jpg]

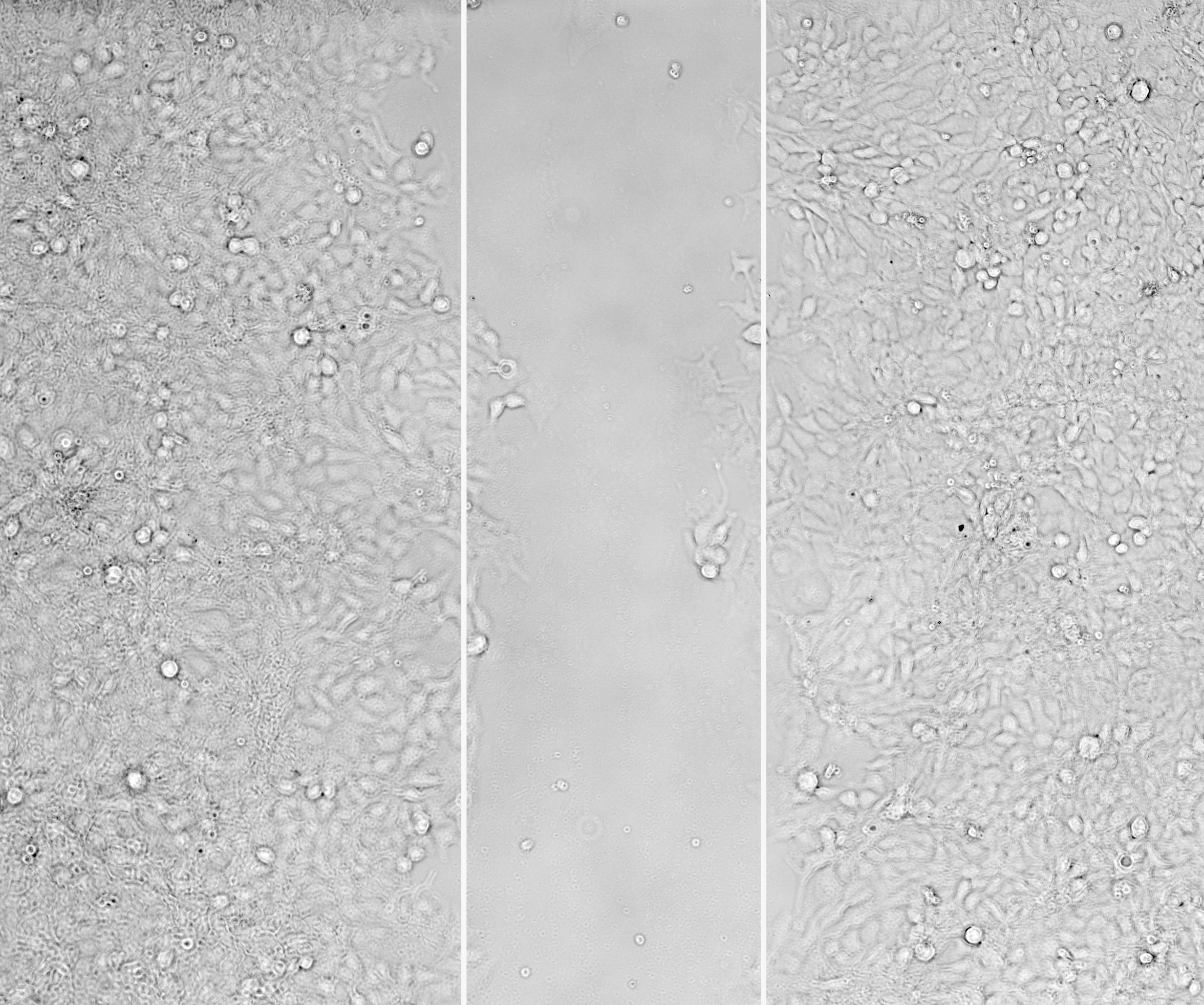

Supplement: Supplementary file 6 — Source Data Fig. 6 [file 44321_2024_33_MOESM6_ESM.zip › Figure 6/6C/ECC1 Glucose/48H-20mM.jpg]

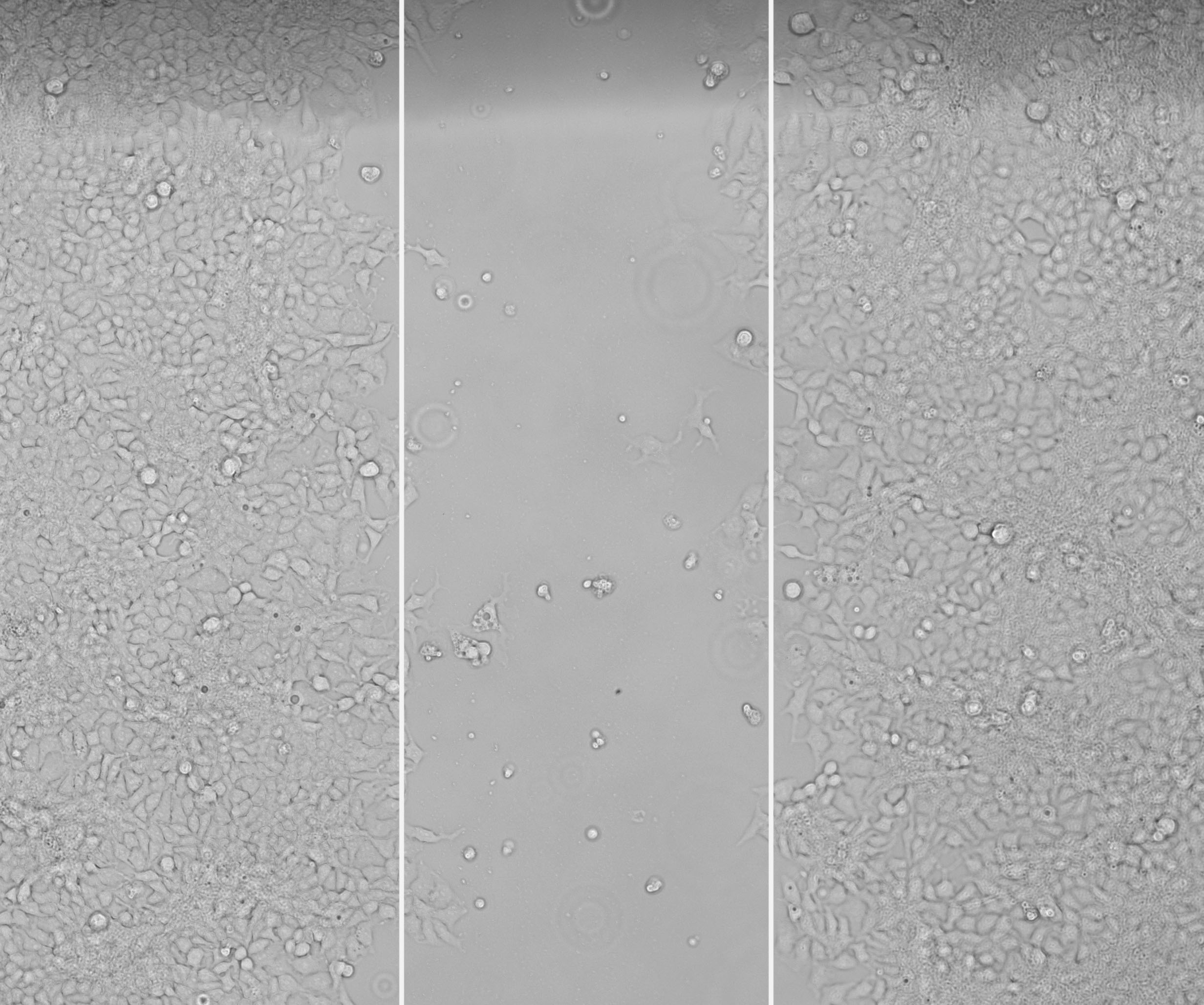

Supplement: Supplementary file 6 — Source Data Fig. 6 [file 44321_2024_33_MOESM6_ESM.zip › Figure 6/6C/ECC1 Glucose/48H-5mM.jpg]

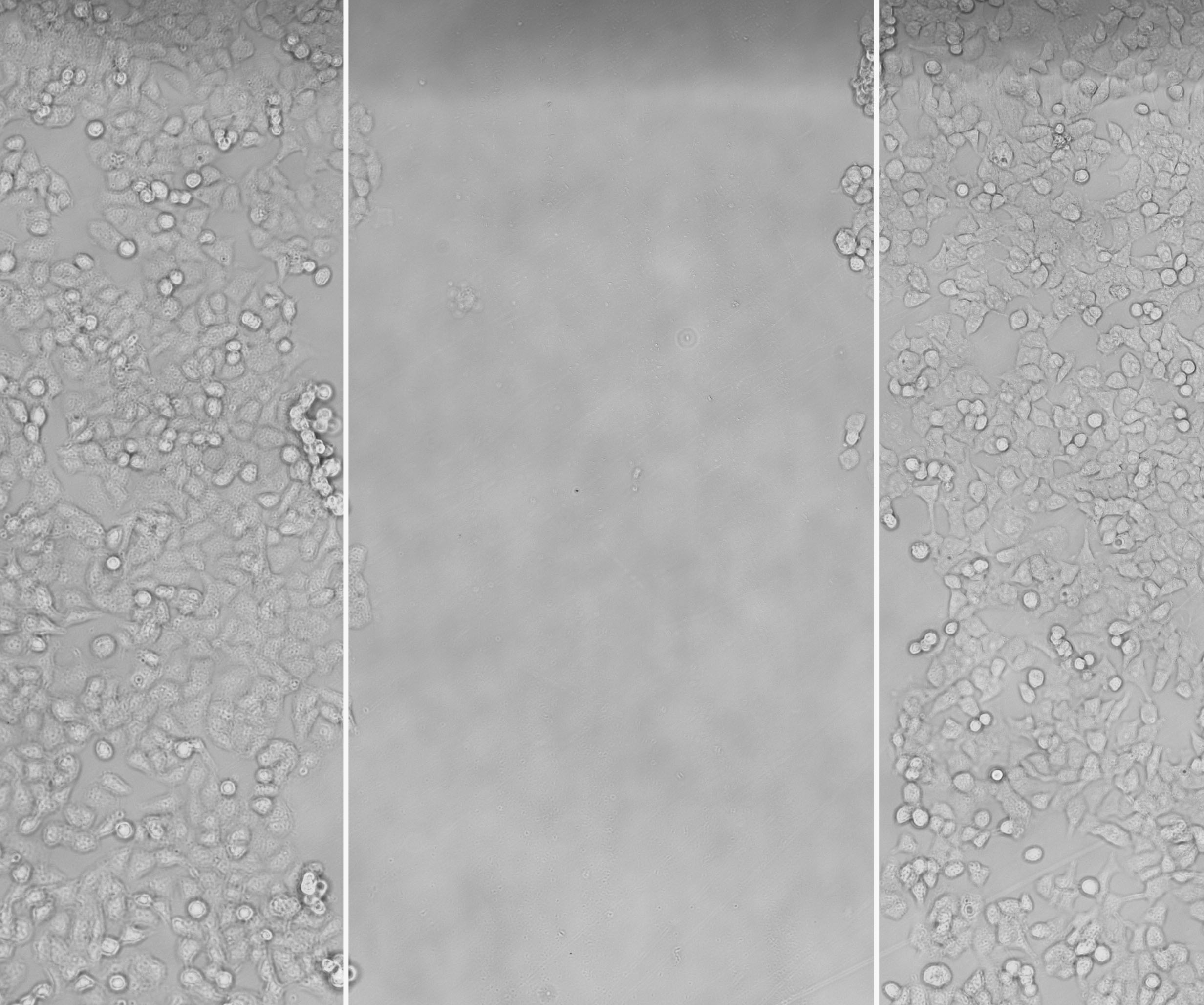

Supplement: Supplementary file 6 — Source Data Fig. 6 [file 44321_2024_33_MOESM6_ESM.zip › Figure 6/6C/ECC1 Glutamine/0H-10mM.jpg]

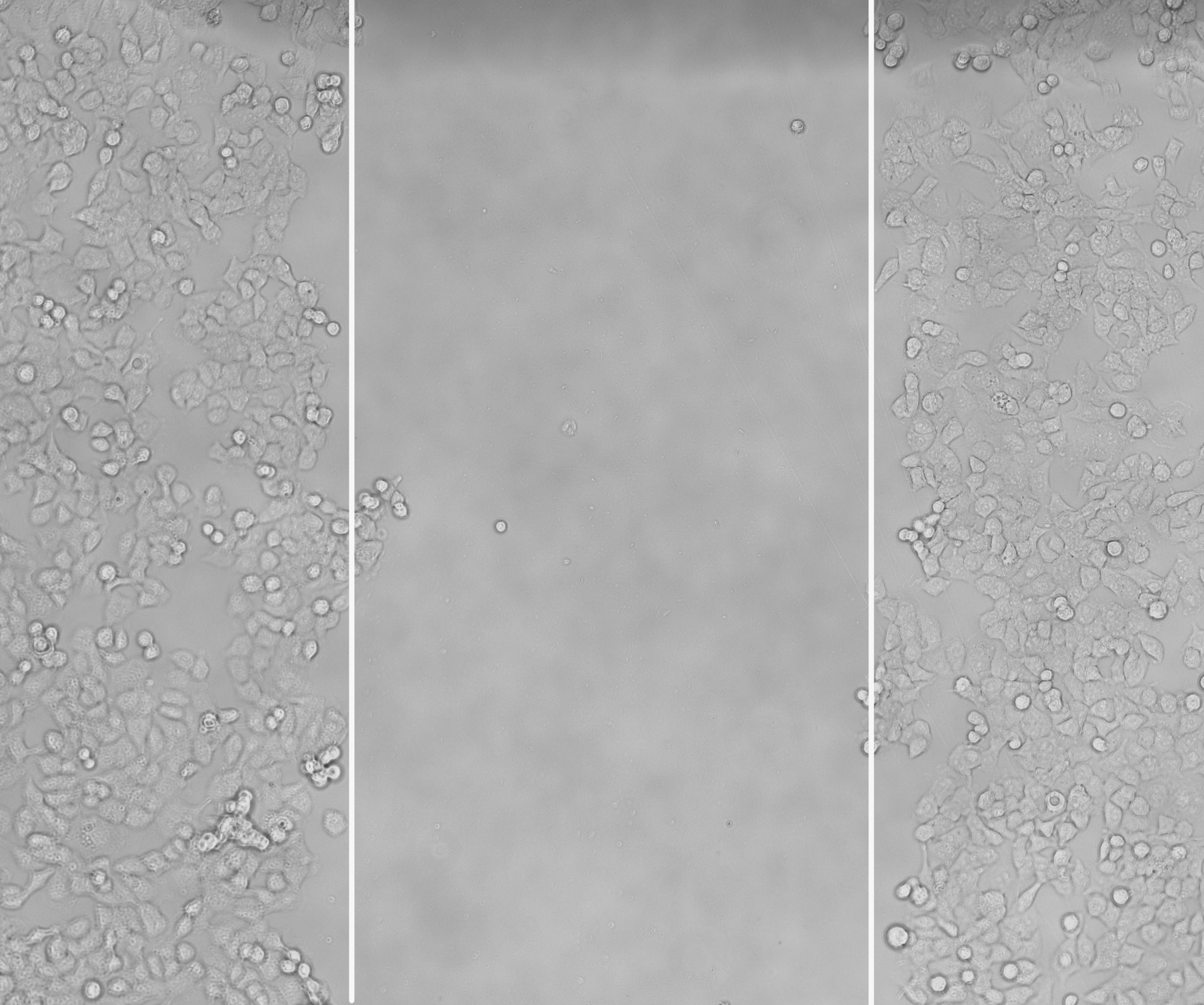

Supplement: Supplementary file 6 — Source Data Fig. 6 [file 44321_2024_33_MOESM6_ESM.zip › Figure 6/6C/ECC1 Glutamine/0H-1mM.jpg]

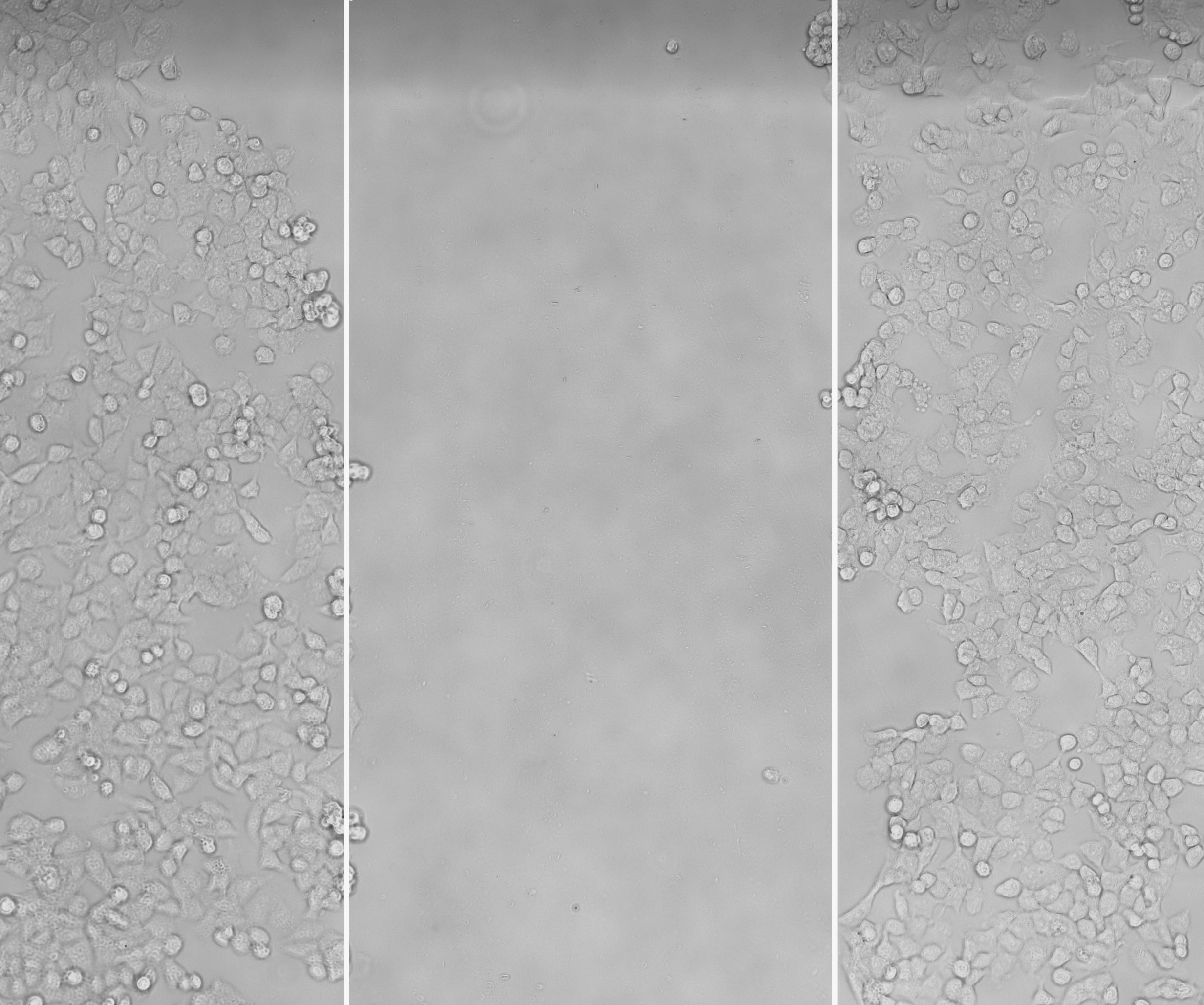

Supplement: Supplementary file 6 — Source Data Fig. 6 [file 44321_2024_33_MOESM6_ESM.zip › Figure 6/6C/ECC1 Glutamine/0H-2mM.jpg]

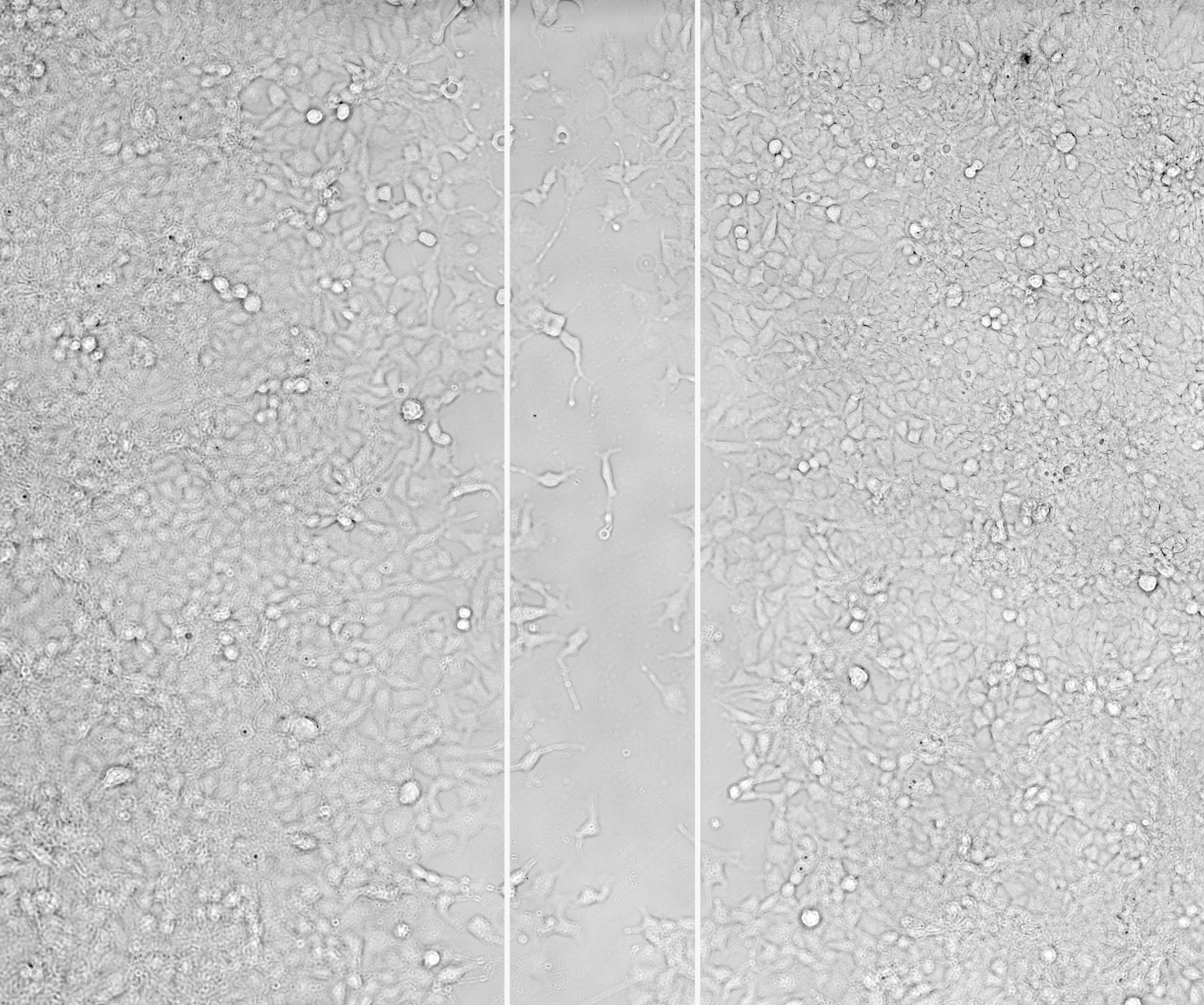

Supplement: Supplementary file 6 — Source Data Fig. 6 [file 44321_2024_33_MOESM6_ESM.zip › Figure 6/6C/ECC1 Glutamine/48H-10mM.jpg]

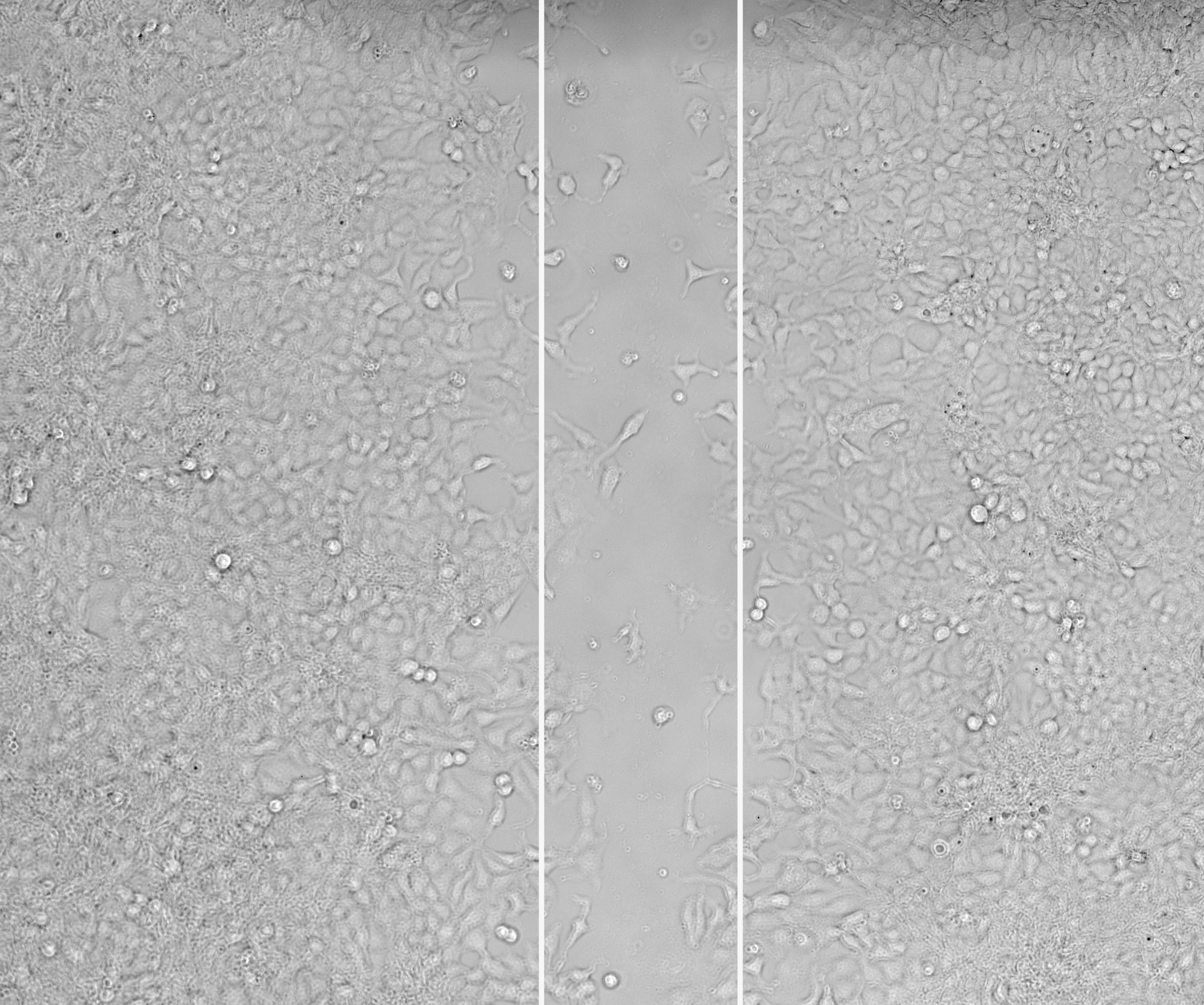

Supplement: Supplementary file 6 — Source Data Fig. 6 [file 44321_2024_33_MOESM6_ESM.zip › Figure 6/6C/ECC1 Glutamine/48H-1mM.jpg]

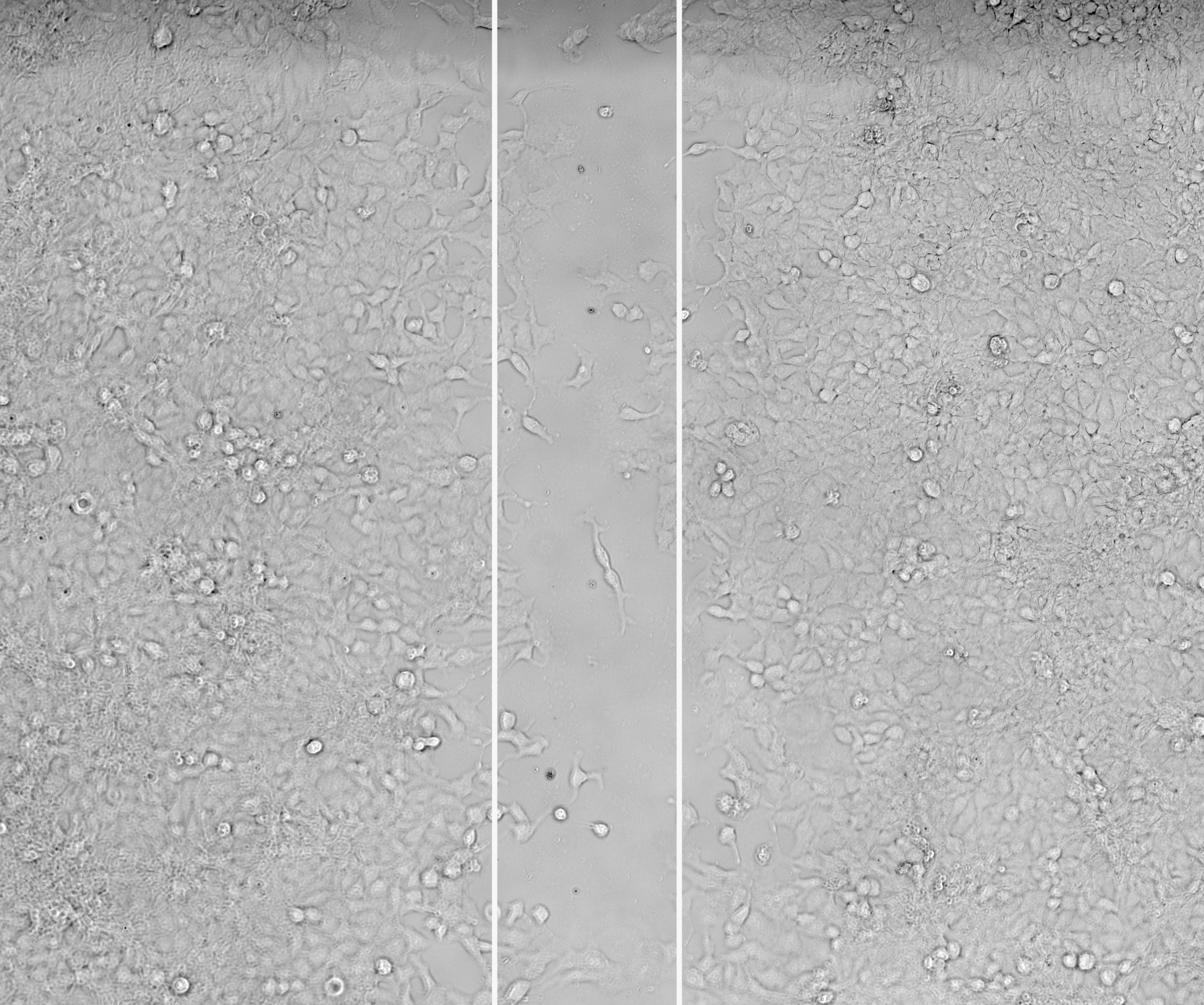

Supplement: Supplementary file 6 — Source Data Fig. 6 [file 44321_2024_33_MOESM6_ESM.zip › Figure 6/6C/ECC1 Glutamine/48H-2mM.jpg]

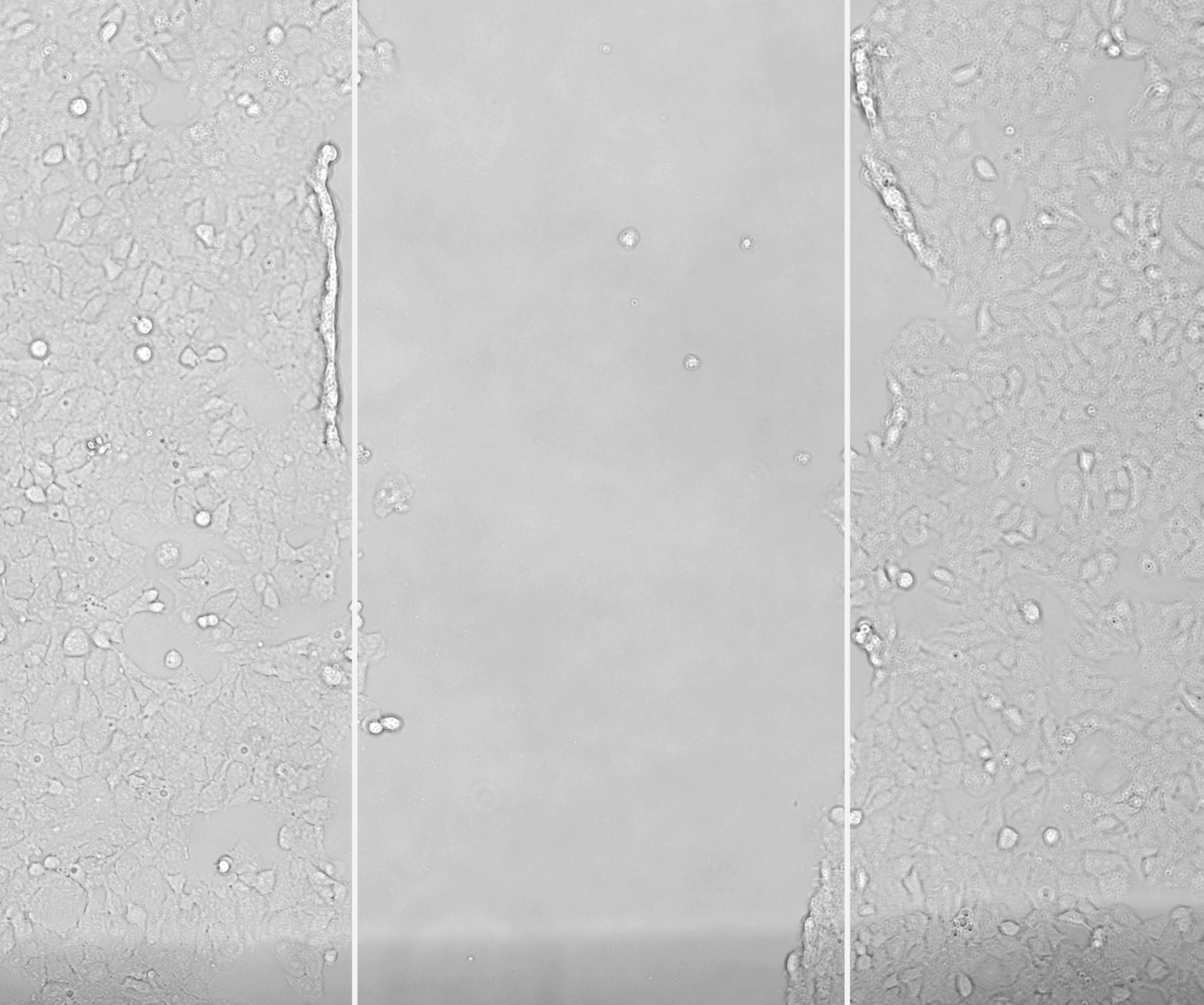

Supplement: Supplementary file 6 — Source Data Fig. 6 [file 44321_2024_33_MOESM6_ESM.zip › Figure 6/6C/Ishikawa Cholesterol linoleate/0H-0μM.jpg]

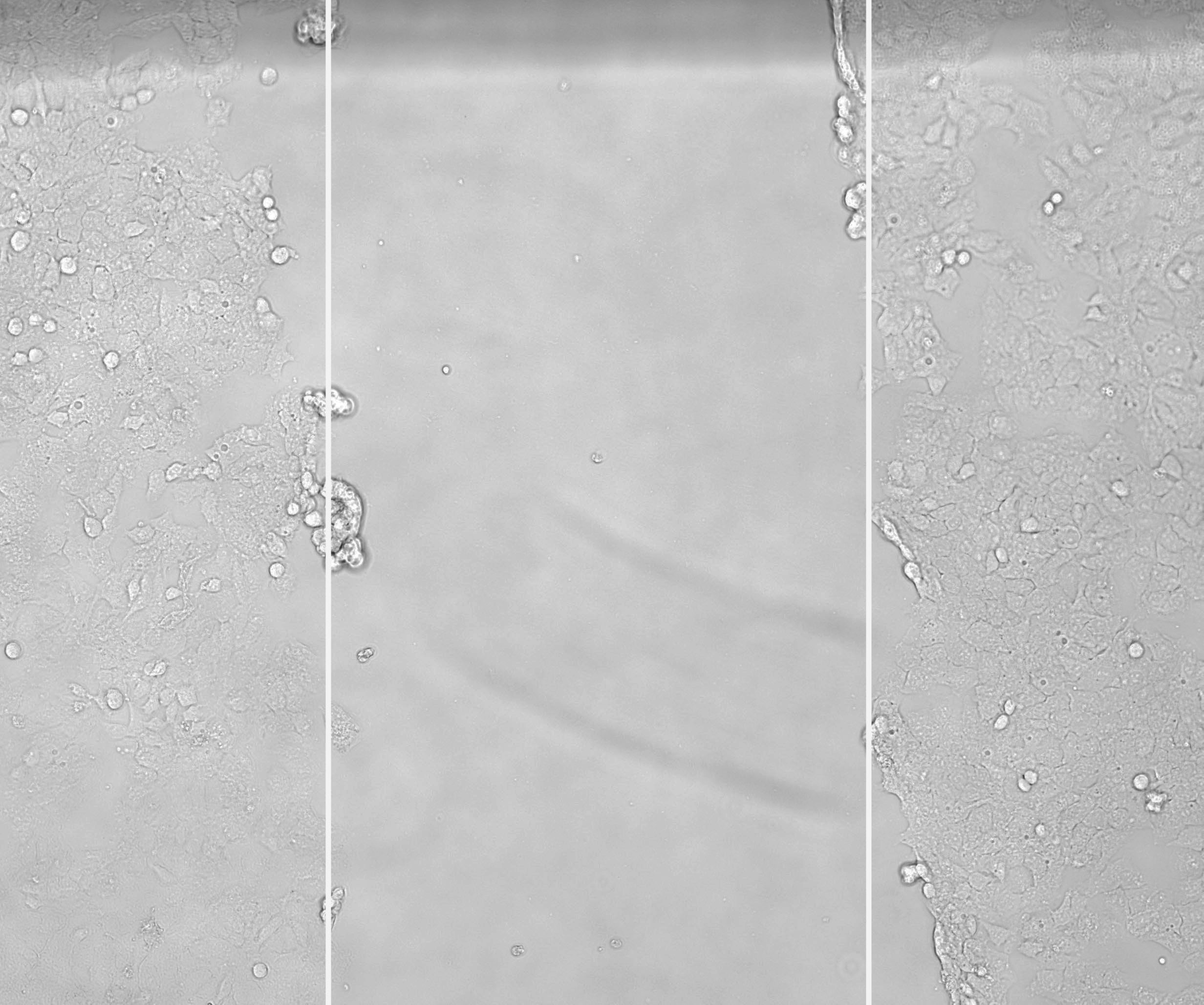

Supplement: Supplementary file 6 — Source Data Fig. 6 [file 44321_2024_33_MOESM6_ESM.zip › Figure 6/6C/Ishikawa Cholesterol linoleate/0H-300μM.jpg]

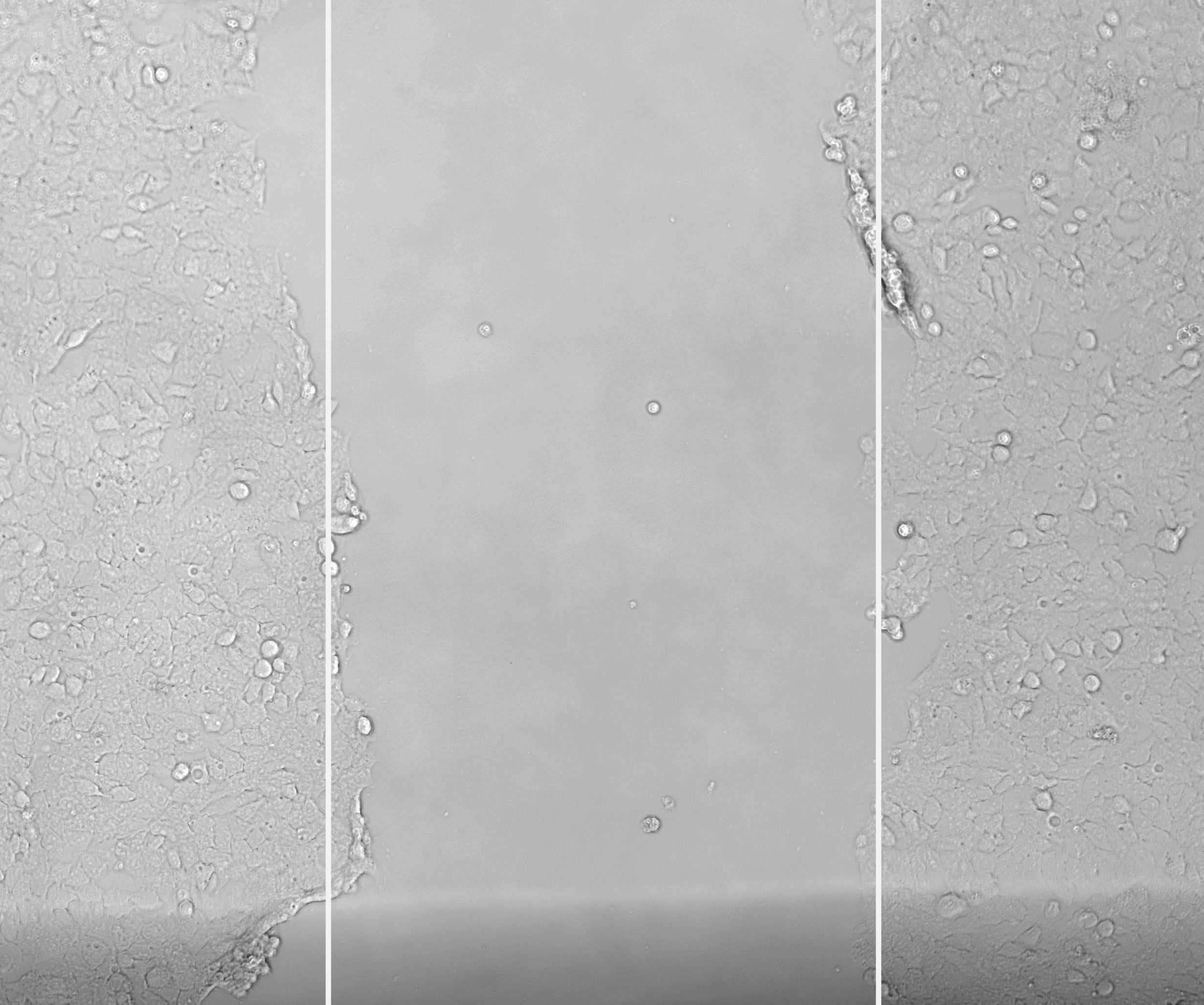

Supplement: Supplementary file 6 — Source Data Fig. 6 [file 44321_2024_33_MOESM6_ESM.zip › Figure 6/6C/Ishikawa Cholesterol linoleate/0H-30μM.jpg]

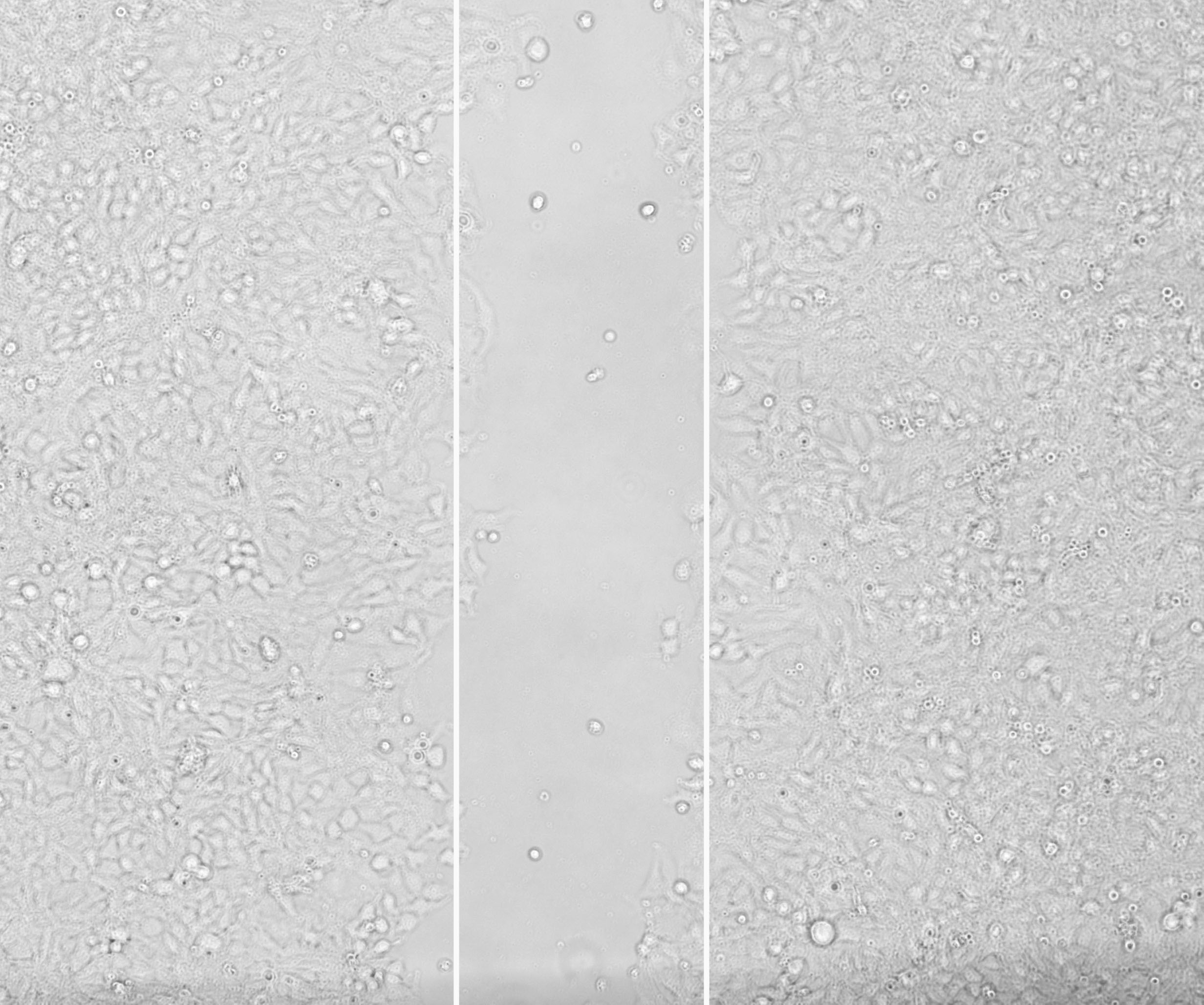

Supplement: Supplementary file 6 — Source Data Fig. 6 [file 44321_2024_33_MOESM6_ESM.zip › Figure 6/6C/Ishikawa Cholesterol linoleate/48H-0μM.jpg]

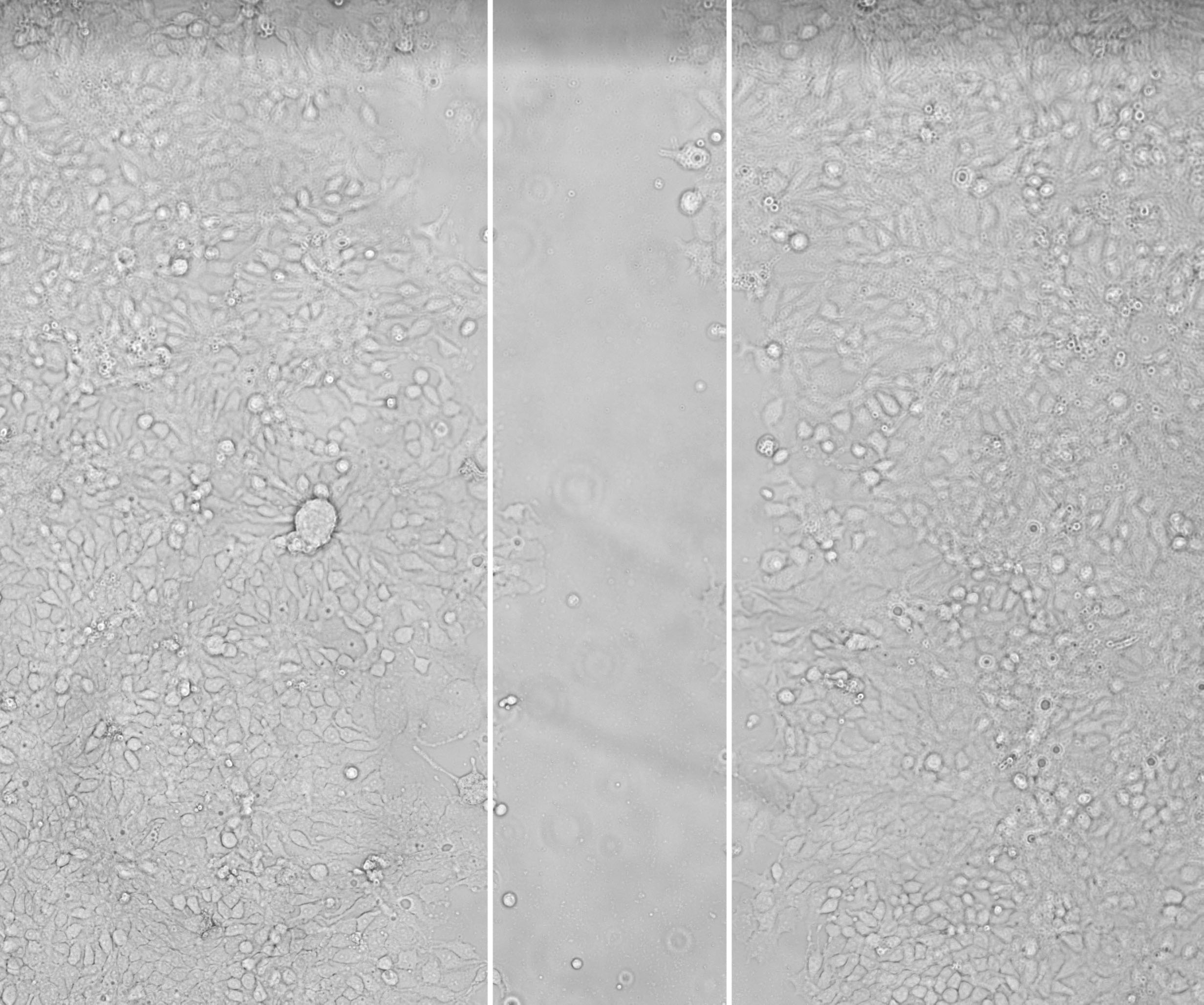

Supplement: Supplementary file 6 — Source Data Fig. 6 [file 44321_2024_33_MOESM6_ESM.zip › Figure 6/6C/Ishikawa Cholesterol linoleate/48H-300μM.jpg]

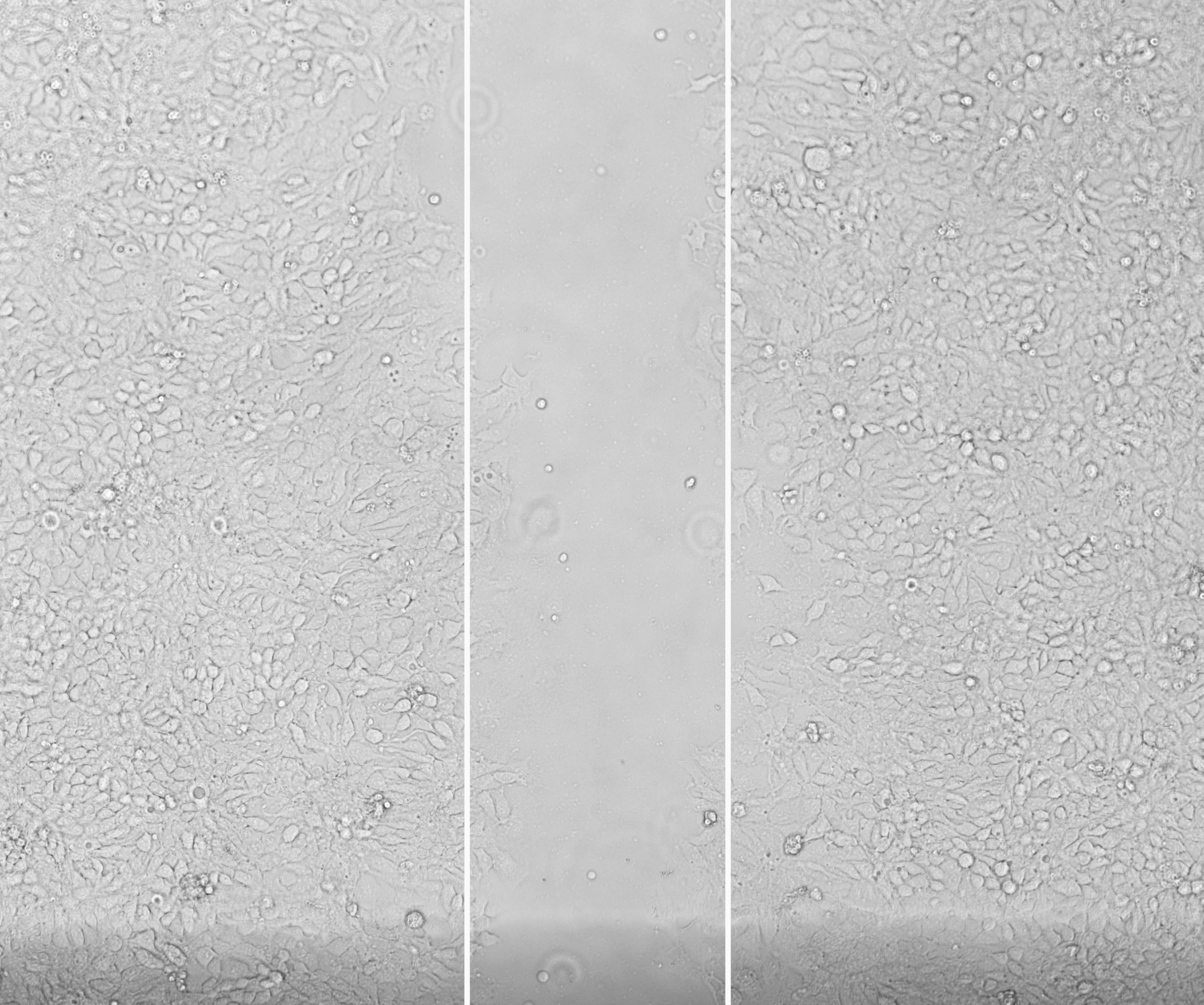

Supplement: Supplementary file 6 — Source Data Fig. 6 [file 44321_2024_33_MOESM6_ESM.zip › Figure 6/6C/Ishikawa Cholesterol linoleate/48H-30μM.jpg]

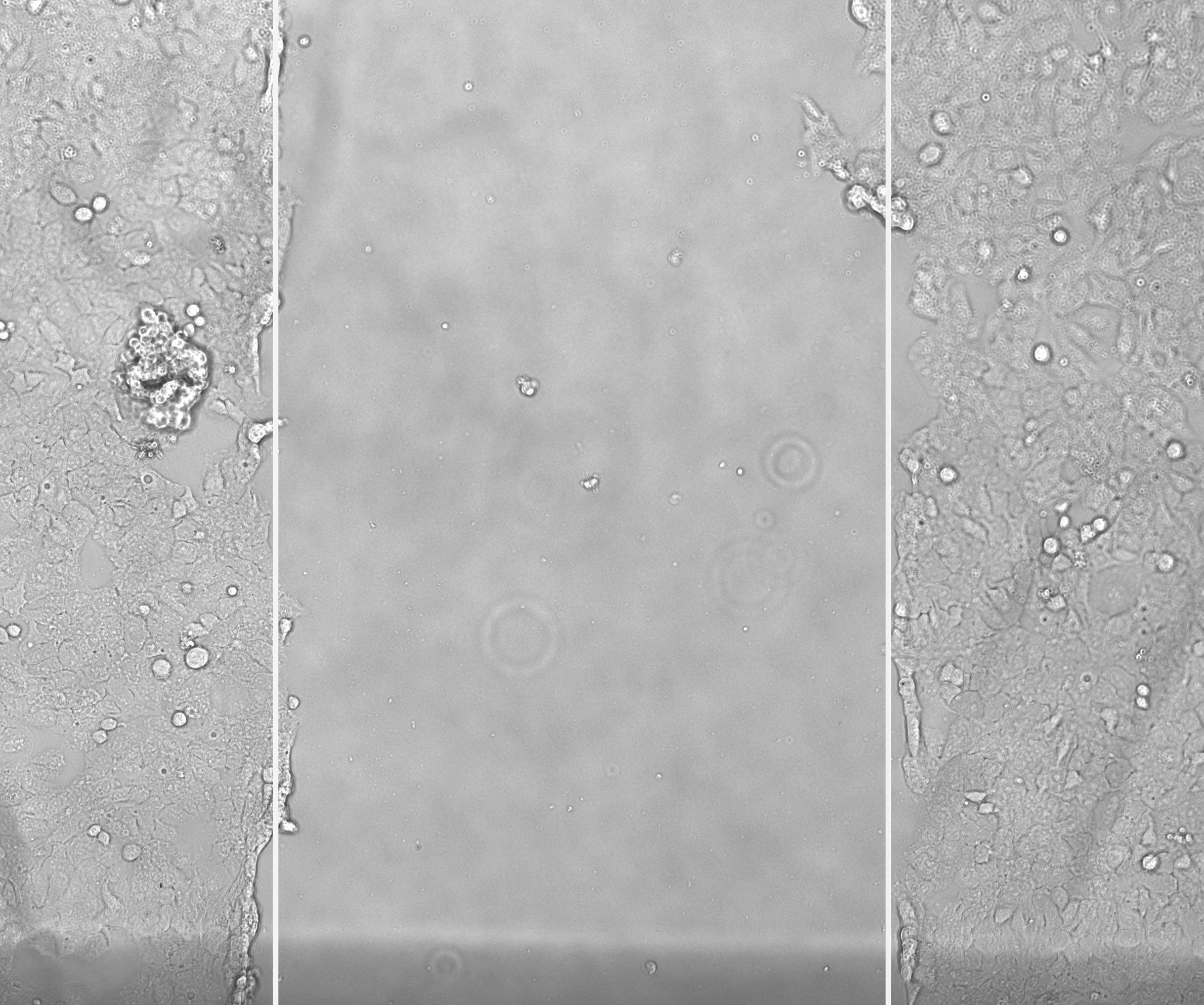

Supplement: Supplementary file 6 — Source Data Fig. 6 [file 44321_2024_33_MOESM6_ESM.zip › Figure 6/6C/Ishikawa Glucose/0H-10mM.jpg]

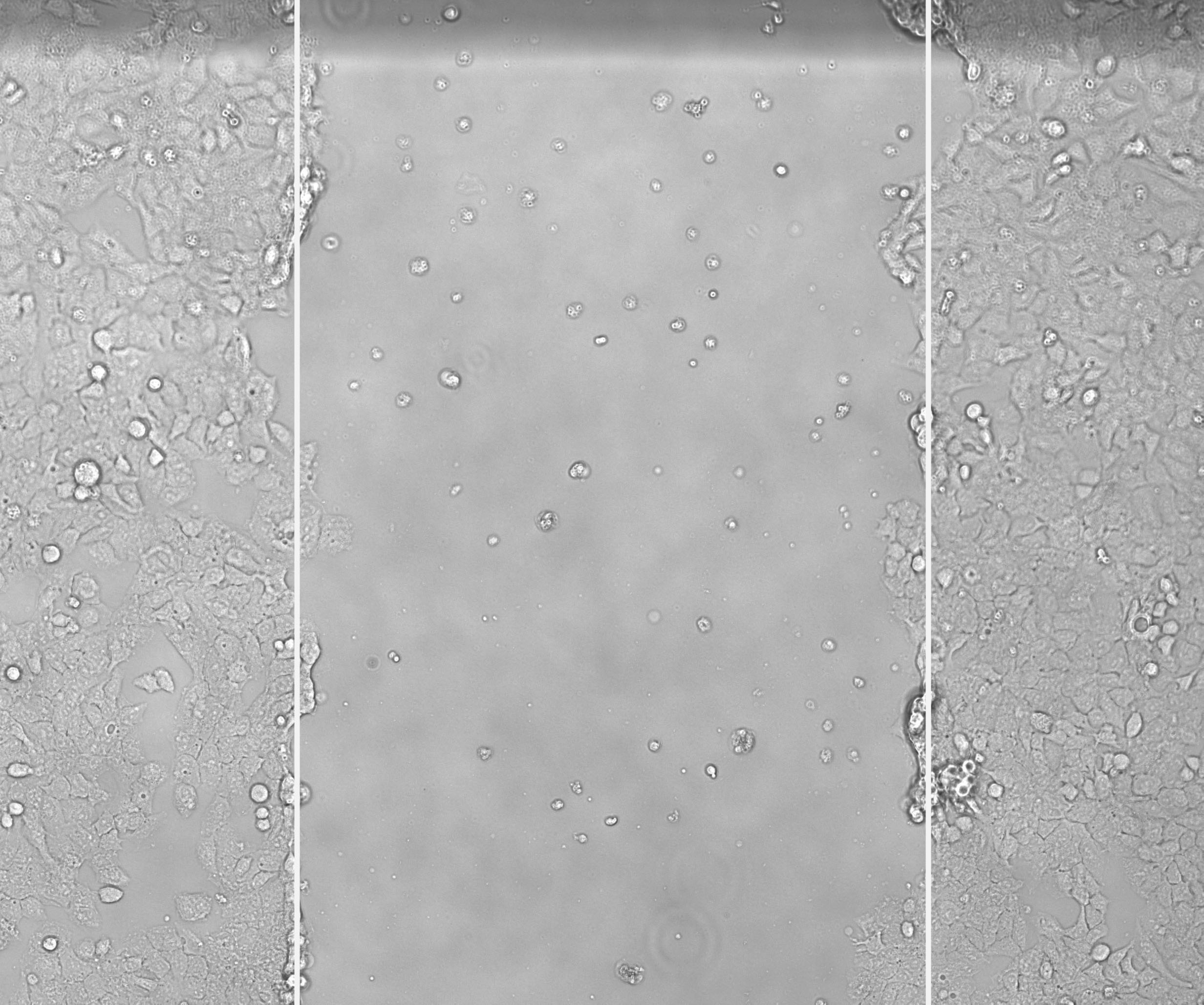

Supplement: Supplementary file 6 — Source Data Fig. 6 [file 44321_2024_33_MOESM6_ESM.zip › Figure 6/6C/Ishikawa Glucose/0H-20mM.jpg]

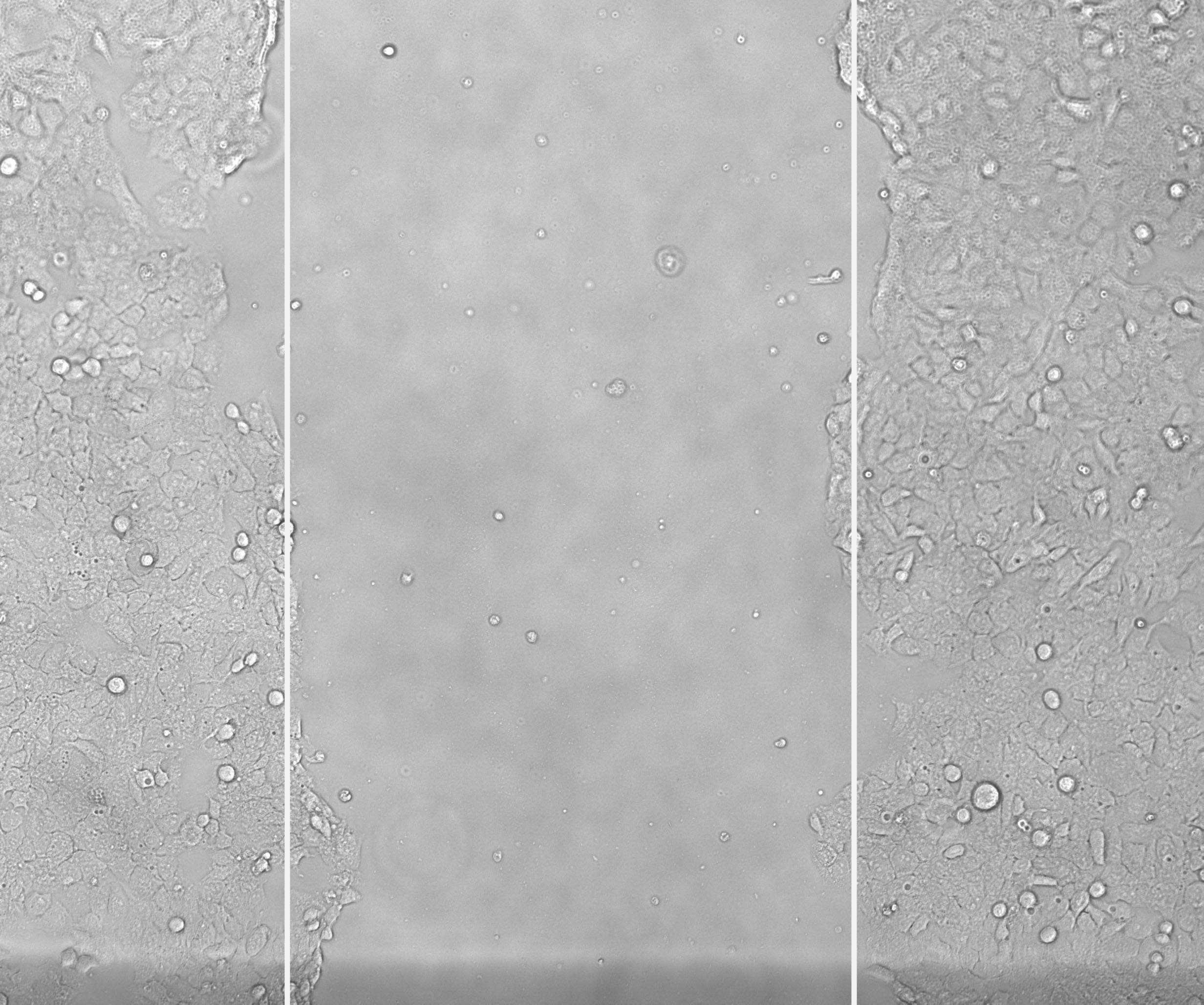

Supplement: Supplementary file 6 — Source Data Fig. 6 [file 44321_2024_33_MOESM6_ESM.zip › Figure 6/6C/Ishikawa Glucose/0H-5mM.jpg]

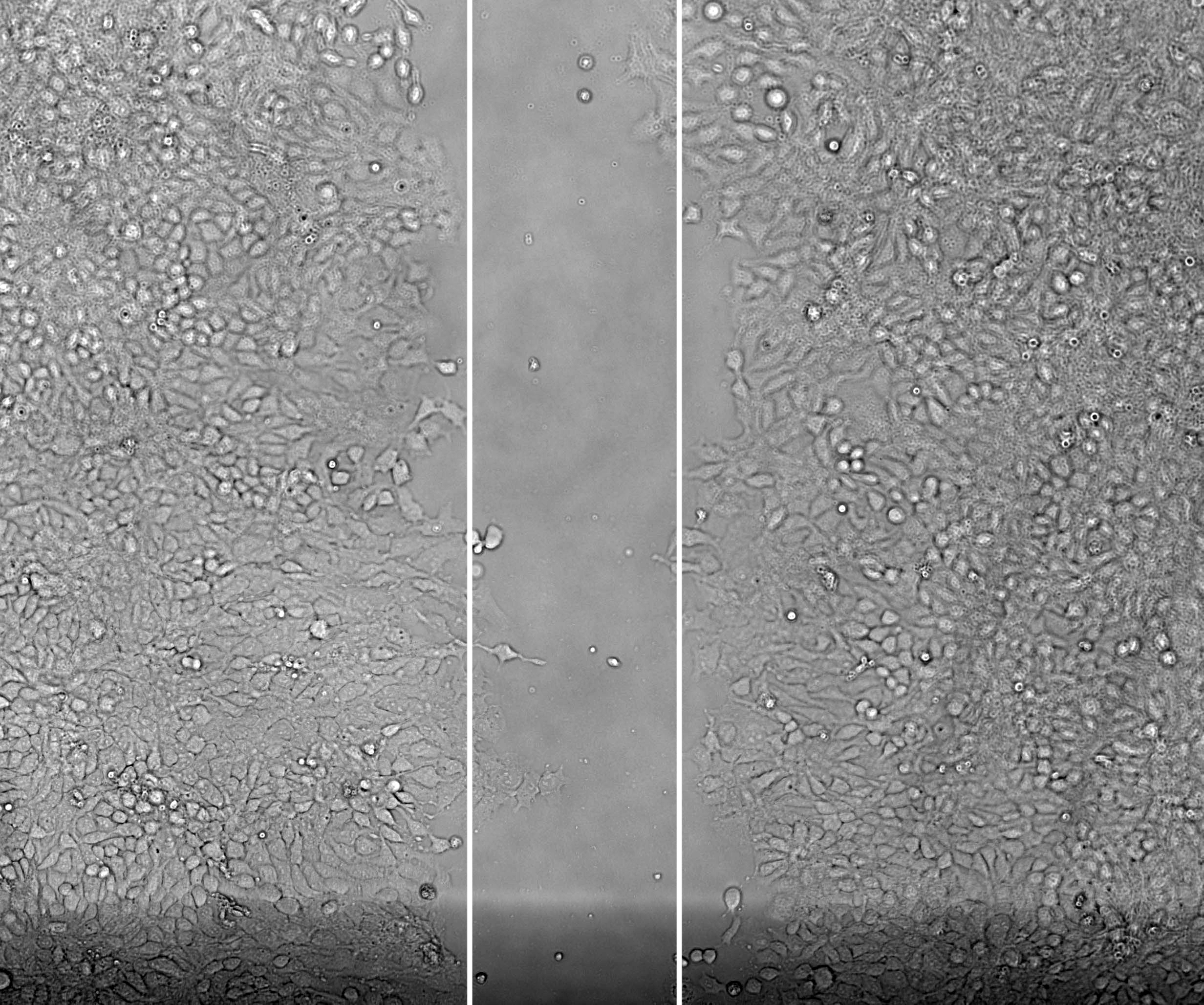

Supplement: Supplementary file 6 — Source Data Fig. 6 [file 44321_2024_33_MOESM6_ESM.zip › Figure 6/6C/Ishikawa Glucose/48H-10mM.jpg]

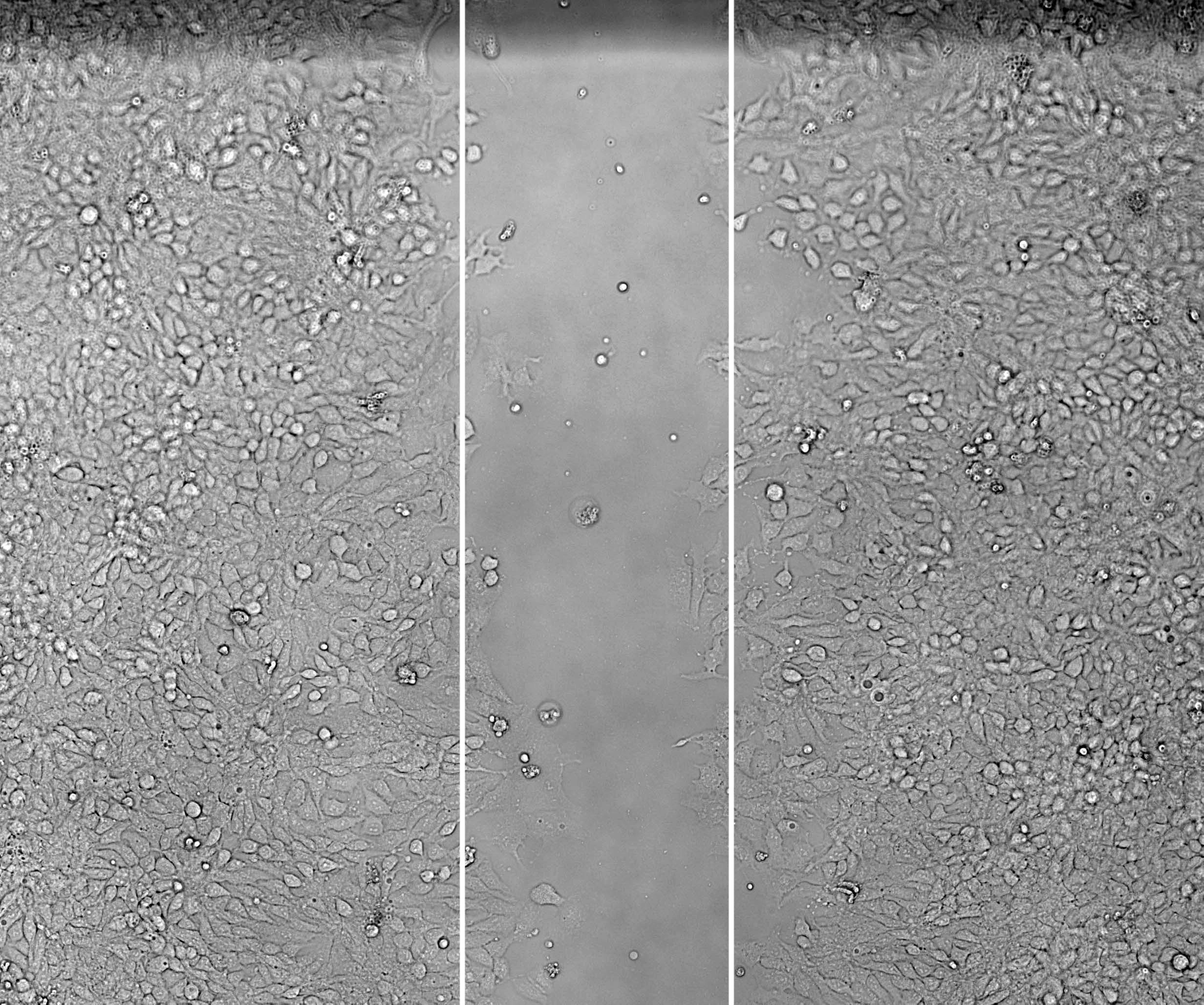

Supplement: Supplementary file 6 — Source Data Fig. 6 [file 44321_2024_33_MOESM6_ESM.zip › Figure 6/6C/Ishikawa Glucose/48H-20mM.jpg]

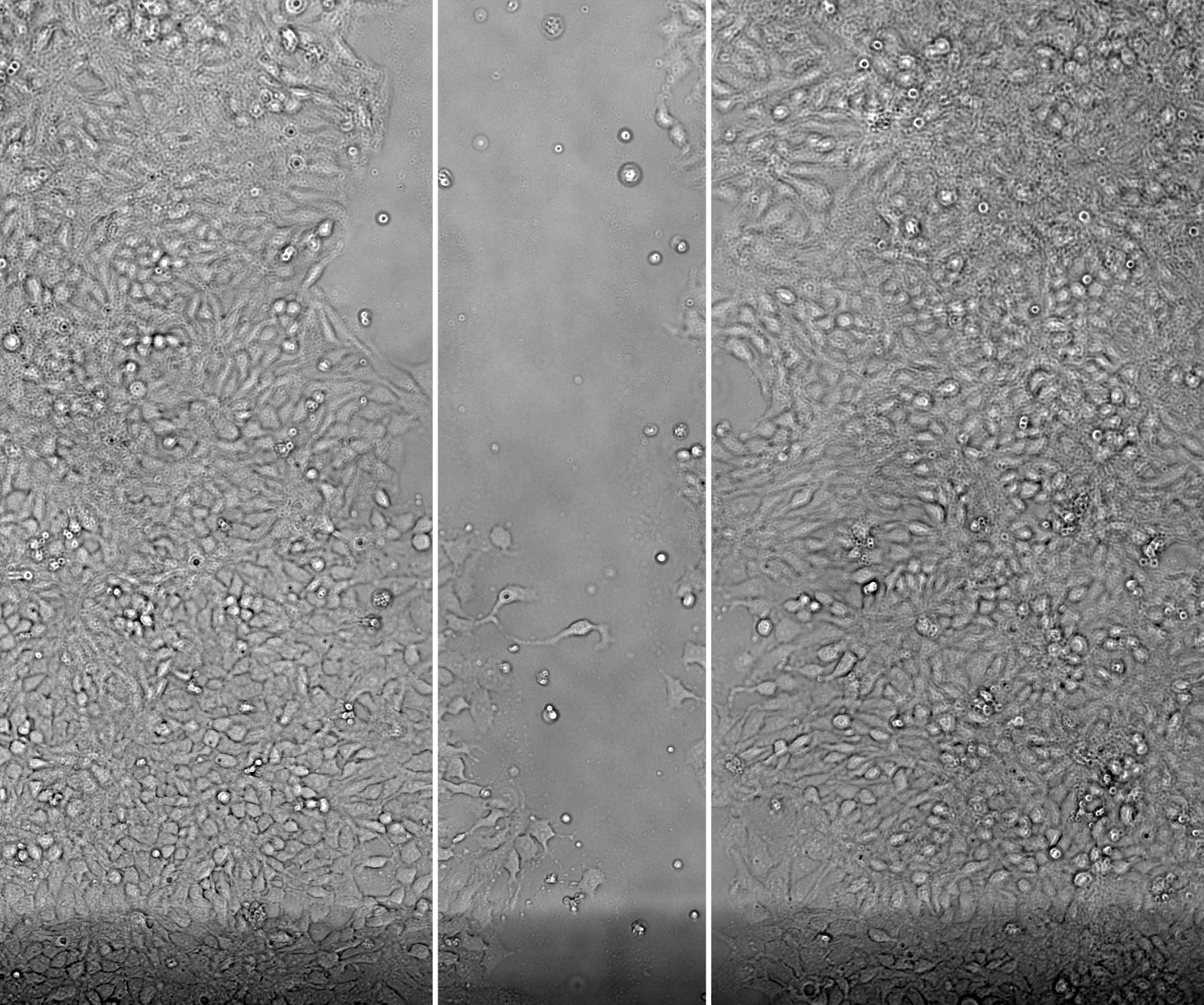

Supplement: Supplementary file 6 — Source Data Fig. 6 [file 44321_2024_33_MOESM6_ESM.zip › Figure 6/6C/Ishikawa Glucose/48H-5mM.jpg]

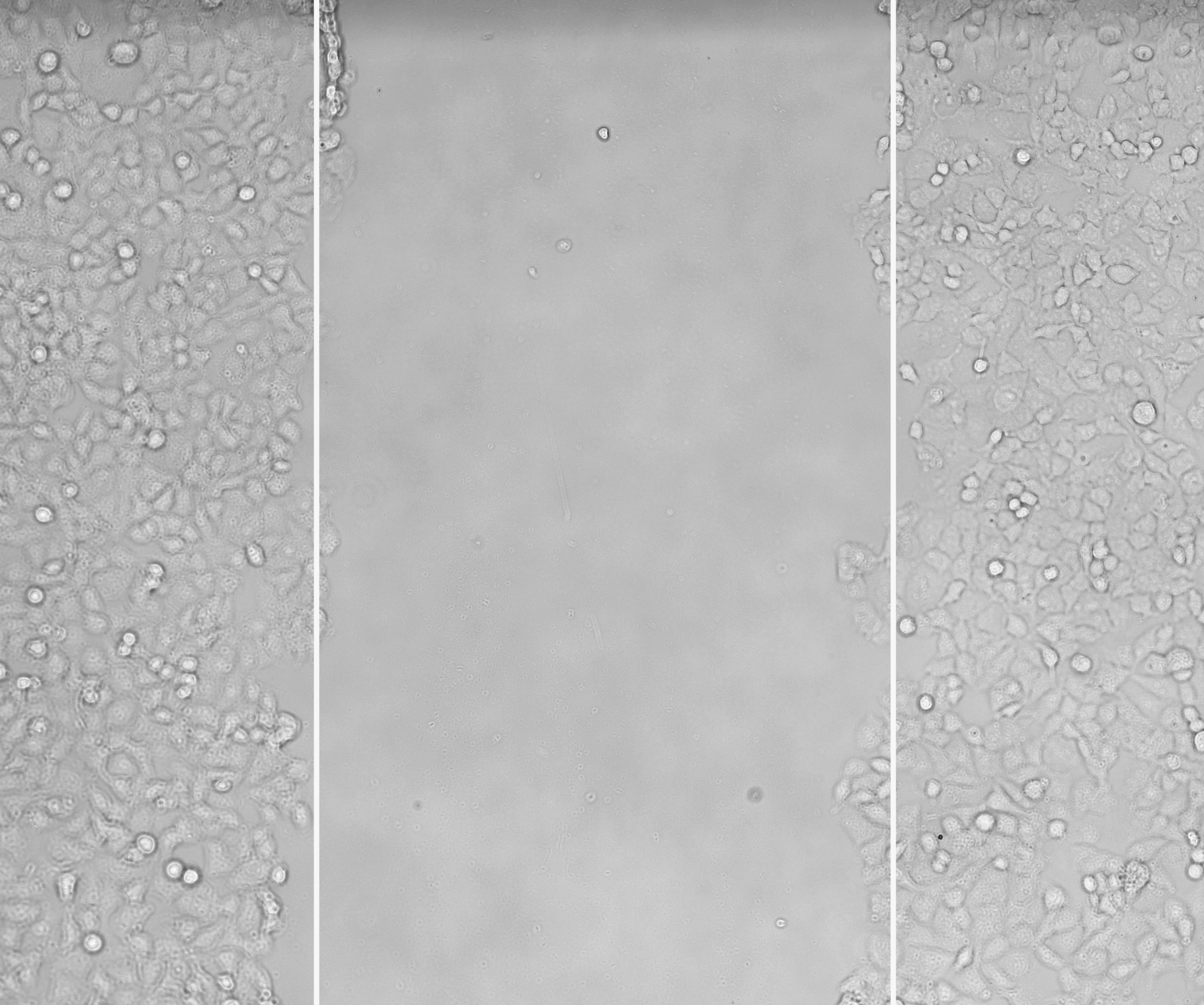

Supplement: Supplementary file 6 — Source Data Fig. 6 [file 44321_2024_33_MOESM6_ESM.zip › Figure 6/6C/Ishikawa Glutamine/0H-10mM.jpg]

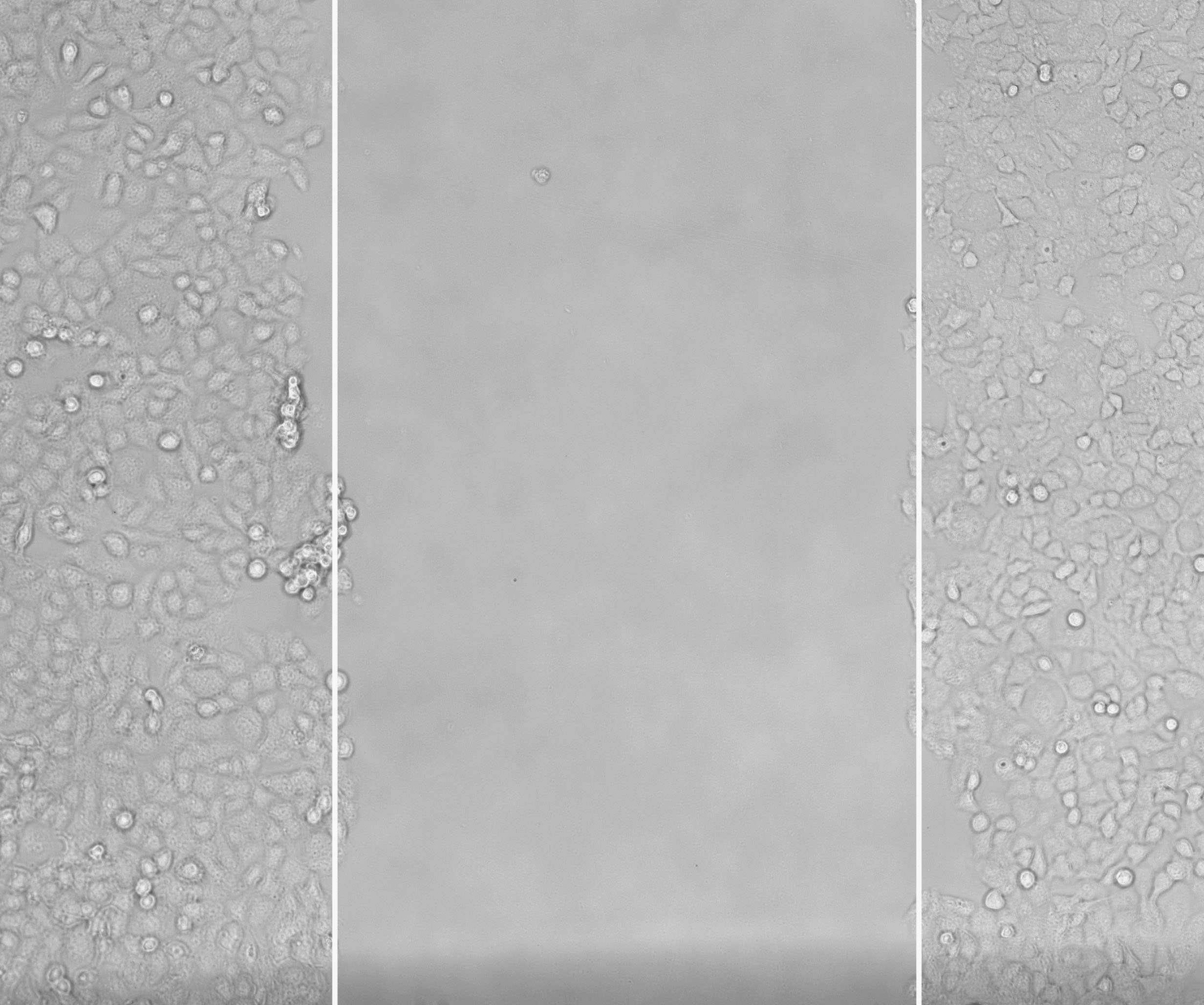

Supplement: Supplementary file 6 — Source Data Fig. 6 [file 44321_2024_33_MOESM6_ESM.zip › Figure 6/6C/Ishikawa Glutamine/0H-1mM.jpg]

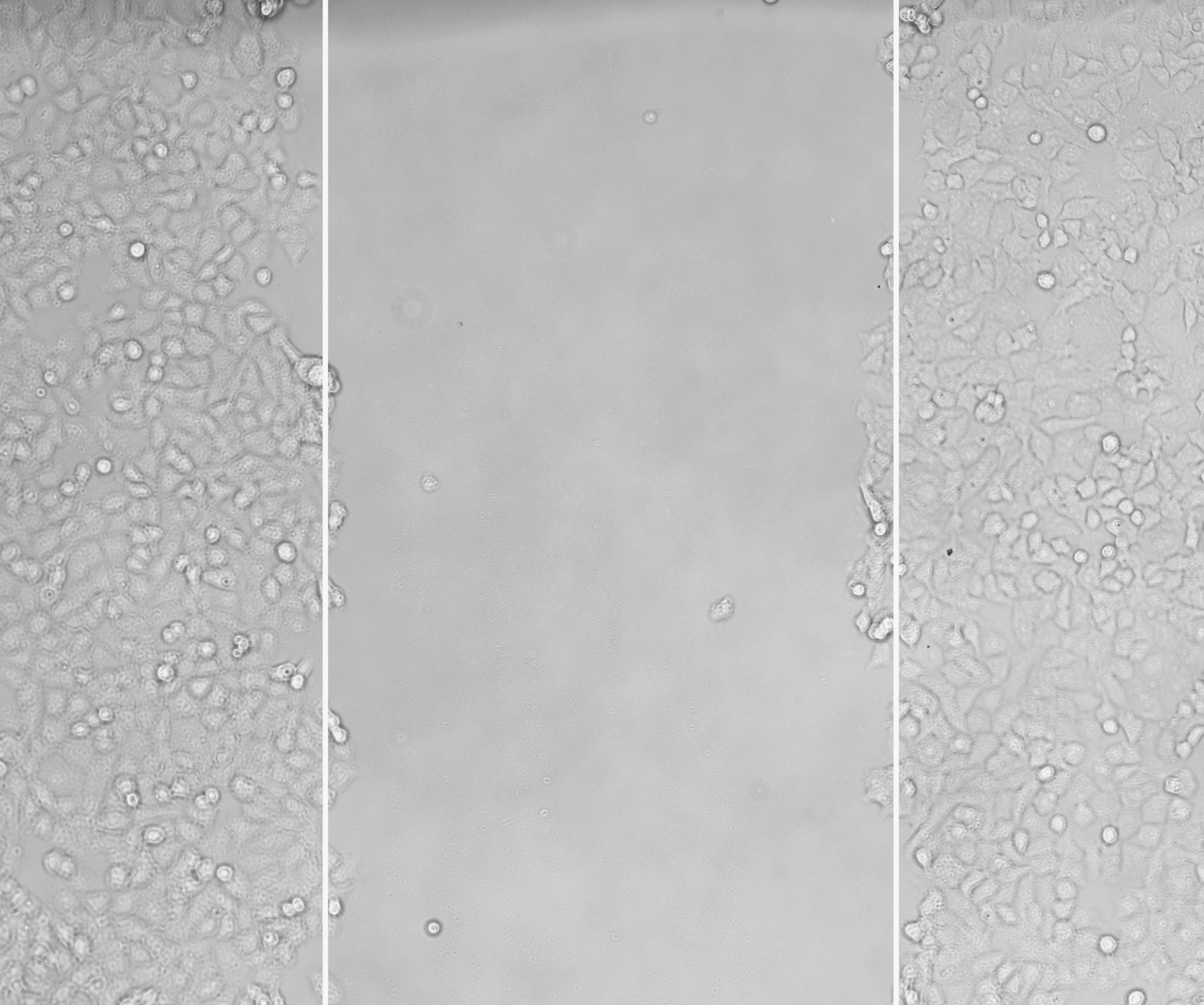

Supplement: Supplementary file 6 — Source Data Fig. 6 [file 44321_2024_33_MOESM6_ESM.zip › Figure 6/6C/Ishikawa Glutamine/0H-2mM.jpg]

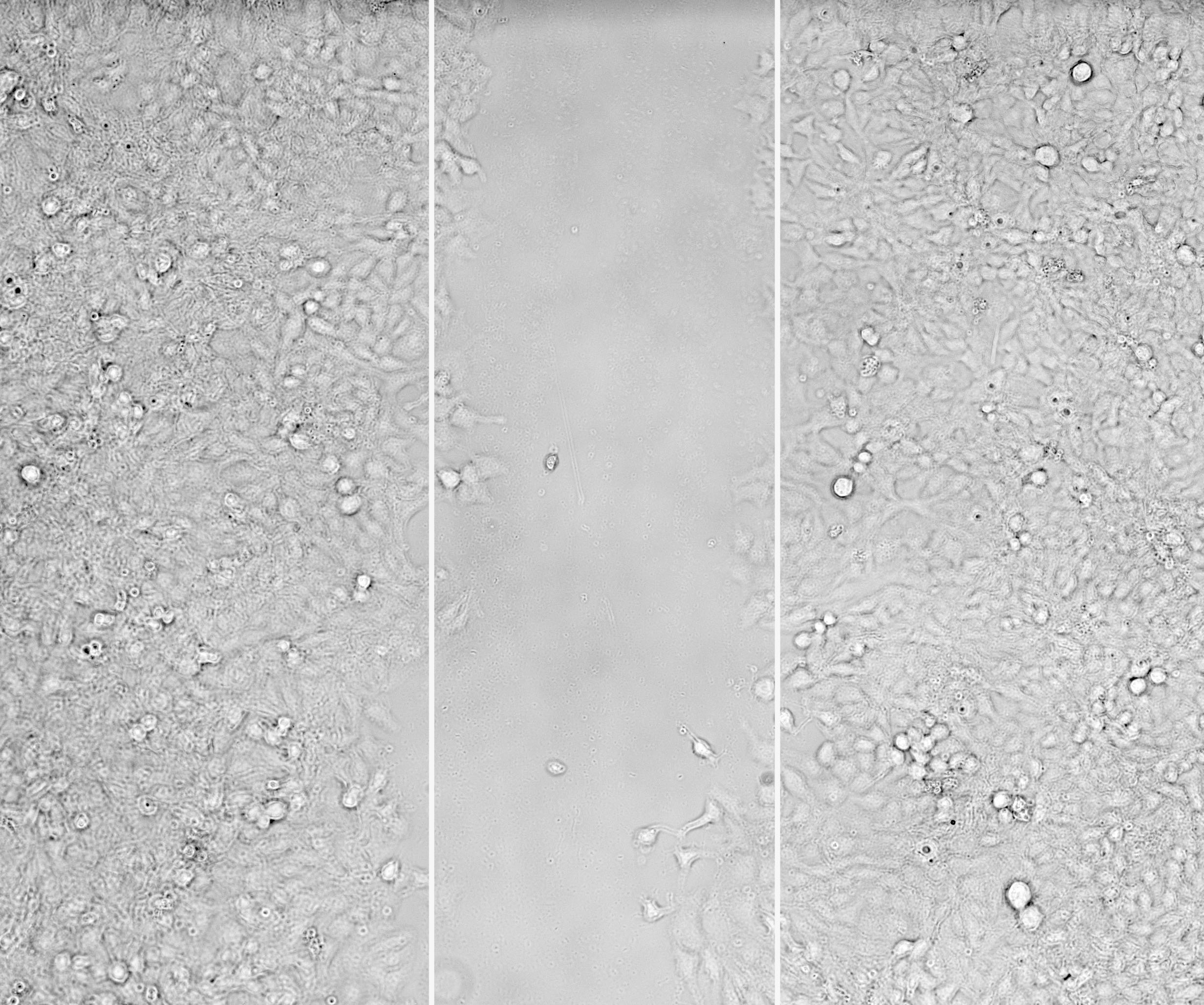

Supplement: Supplementary file 6 — Source Data Fig. 6 [file 44321_2024_33_MOESM6_ESM.zip › Figure 6/6C/Ishikawa Glutamine/48H-10mM.jpg]

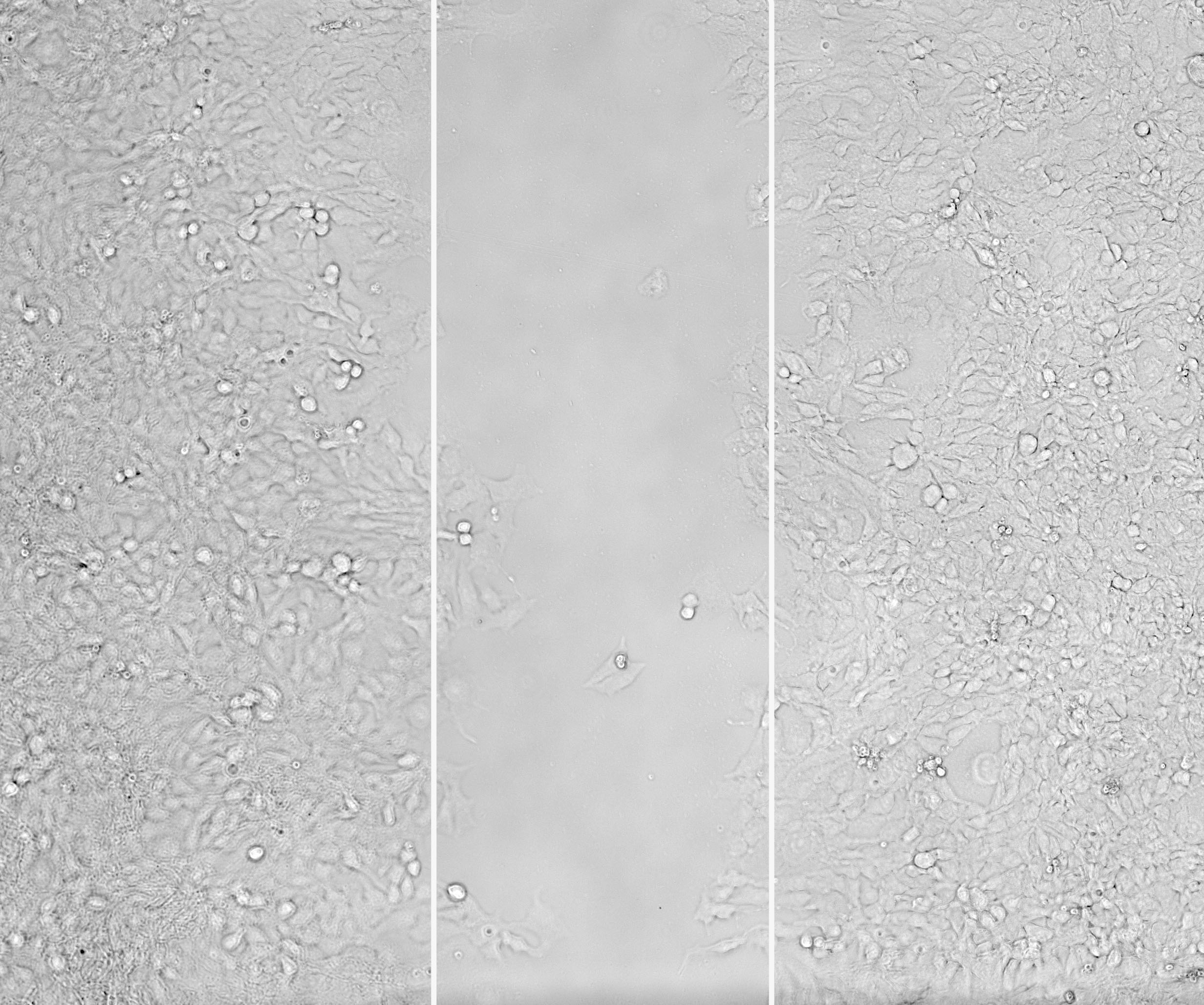

Supplement: Supplementary file 6 — Source Data Fig. 6 [file 44321_2024_33_MOESM6_ESM.zip › Figure 6/6C/Ishikawa Glutamine/48H-1mM.jpg]

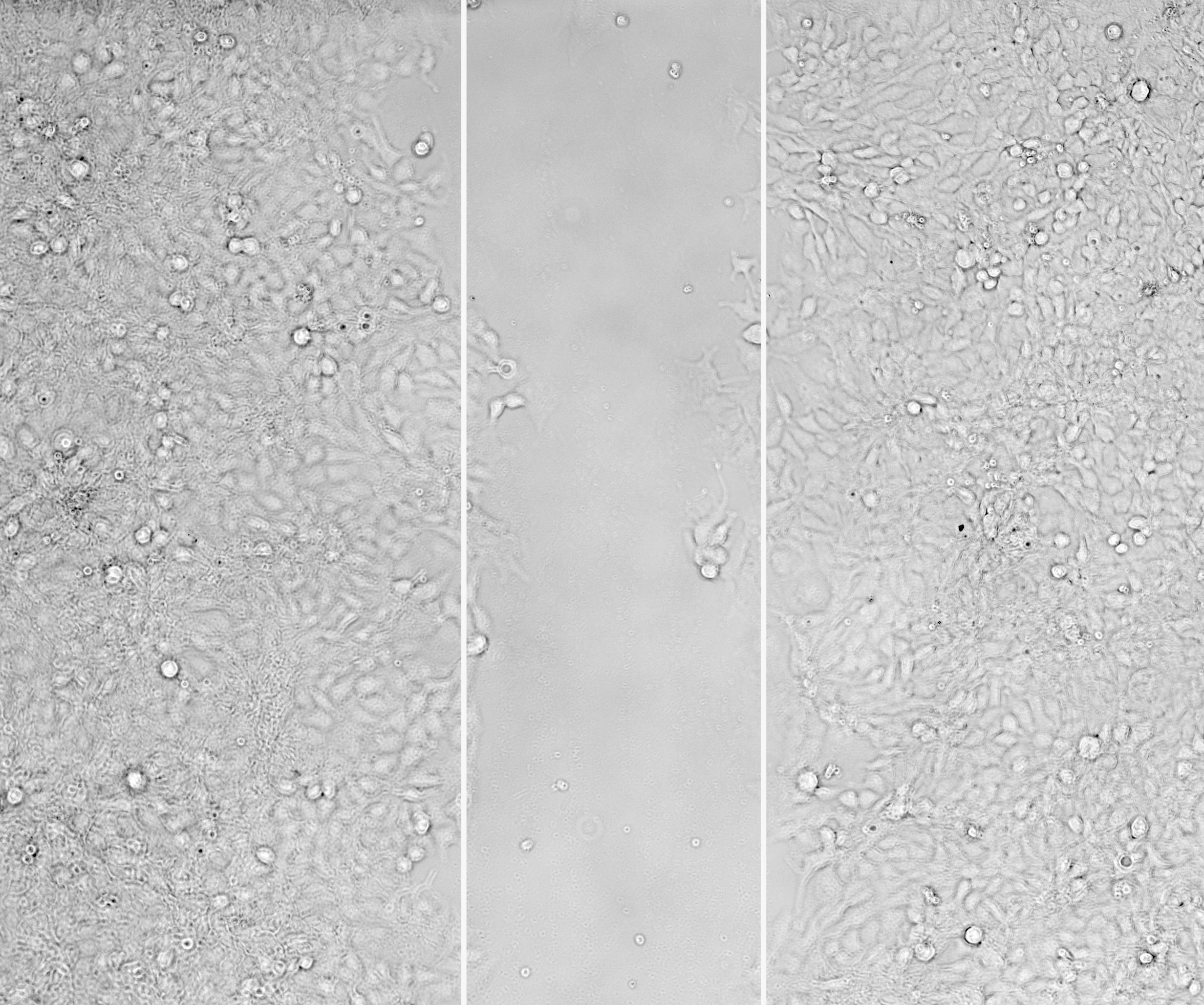

Supplement: Supplementary file 6 — Source Data Fig. 6 [file 44321_2024_33_MOESM6_ESM.zip › Figure 6/6C/Ishikawa Glutamine/48H-2mM.jpg]
